# A primer on Variational Laplace (VL)

**DOI:** 10.1016/j.neuroimage.2023.120310

**Published:** 2023-10-01

**Authors:** Peter Zeidman, Karl Friston, Thomas Parr

**Affiliations:** Wellcome Centre for Human Neuroimaging, UCL, 12 Queen Square, London WC1N 3AR, United Kingdom

**Keywords:** Variational Laplace, Bayes, DCM, Modelling

## Abstract

This article details a scheme for approximate Bayesian inference, which has underpinned thousands of neuroimaging studies since its introduction 15 years ago. Variational Laplace (VL) provides a generic approach to fitting linear or non-linear models, which may be static or dynamic, returning a posterior probability density over the model parameters and an approximation of log model evidence, which enables Bayesian model comparison. VL applies variational Bayesian inference in conjunction with quadratic or Laplace approximations of the evidence lower bound (free energy). Importantly, update equations do not need to be derived for each model under consideration, providing a general method for fitting a broad class of models. This primer is intended for experimenters and modellers who may wish to fit models to data using variational Bayesian methods, without assuming previous experience of variational Bayes or machine learning. Accompanying code demonstrates how to fit different kinds of model using the reference implementation of the VL scheme in the open-source Statistical Parametric Mapping (SPM) software package. In addition, we provide a standalone software function that does not require SPM, in order to ease translation to other fields, together with detailed pseudocode. Finally, the supplementary materials provide worked derivations of the key equations.

## Highlights

• Variational Laplace (VL) is a scheme for Bayesian modelling.
• VL is widely used in neuroimaging, in particular DCM.
• This paper provides a tutorial explanation of the math and algorithms.
• New standalone code is provided to enable re-implementation.
• The supplementary materials provide worked derivations.

## 1 Introduction

Scientific enquiry typically involves inferring quantities that cannot be directly observed. For example, a neuroscientist may wish to investigate the activity of neural populations from electrical activity that can be measured on the scalp. Similarly, a seismologist may wish to make inferences about geophysical events that occur beneath the surface of the earth, from seismograph measurements taken at the surface. Both of these are examples of *ill-posed problems*, where multiple configurations of the underlying system of interest could lead to similar measurements. Consequently, any inferences that are made about the underlying mechanisms that generate the data will typically involve uncertainty. This uncertainty needs to be quantified when drawing conclusions, which is why probabilistic, or *Bayesian* inference methods are typically used.

This article explains the mathematics behind a scheme for Bayesian modelling called Variational Laplace (VL), which is widely used in neuroimaging. It is used to test the statistical evidence for competing models, and in tandem, to rapidly estimates models’ parameters, without the need for computationally demanding sampling procedures found in other Bayesian approaches. VL is the cornerstone of Dynamic Causal Modelling (DCM) for fMRI and M/EEG - a framework for inferring neural connectivity from non-invasive neuroimaging data (Friston et al., 2003). A special case of VL, referred to as variational Restricted Maximum Likelihood (REML), is applied behind the scenes in every Statistical Parametric Mapping (SPM) analysis for estimating temporal auto-correlation, as well as for Bayesian source localisation with M/EEG data (Friston et al., 2008a). The original implementation of VL was in the SPM software package (Friston et al., 2007), and variants of the scheme are now provided in other packages for analysing neural and psychological data, such as the VBA Toolbox (Daunizeau et al., 2014) and TAPAS (Frässle et al., 2021). It is also increasingly being applied to fields beyond neuroimaging, including theoretical neurobiology (Smith et al., 2022), robotics (Lanillos et al., 2021; Lanillos and van Gerven, 2021) and epidemiology (Friston et al., 2020).

Despite its widespread use, understanding VL can be challenging for the uninitiated; the original description of VL assumed some familiarity with variational methods, statistical physics and Bayesian inference. Here, our aim is didactic. We explain the methodology from first principles, which we anticipate will be particularly useful for people interested in developing new models of neuroimaging data, or new software toolboxes for neuroimaging analysis. Additionally, we hope this article will be useful for experimenters, who wish to gain a deeper understanding of how the analyses they routinely conduct are performed under the hood. Finally, VL may have relevance to other fields, including machine learning and other physical / biological sciences. We have therefore provided generic pseudocode and MATLAB code with this paper, which could ease translation for new applications. This article compliments previous tutorial introductions to variational Bayes in other contexts (Chappell et al., 2016; Ostwald et al., 2014), and technical reports which have set out the mathematics of VL in detail (Daunizeau, 2017; Stephan et al., 2005; Friston et al., 2007; Starke and Ostwald, 2017).

### 1.1 Example problem: modelling neural connectivity

To motivate the use of VL, we consider the following classic fMRI study, which has been used to develop and illustrate many new analysis methods in the decades since it was published. Büchel et al. (1998) investigated area V5 of the visual cortex, which was known to be sensitive to visual motion. While undergoing fMRI, participants viewed white dots on a computer screen, which were either in motion or stationary. On a subset of the trials with motion, participants were instructed to pay attention to the *speed* of the dots’ motion. The authors found that the neural response to visual motion in V5 was enhanced when people paid attention to the speed of the moving dots. Attention also enhanced the neural response in brain regions lower in the visual hierarchy (primary visual cortex) and higher in the hierarchy (superior parietal cortex).

Here, for illustrative purposes, we will use the fMRI data from a single participant from Büchel et al. (1998) to address the following question: which neural connections explain why V5 was sensitive to visual motion? We consider two candidate hypotheses^1^:

• H1: Attention modulated *bottom-up* connectivity from primary visual cortex (V1) to V5
• H2: Attention modulated *top-down* connectivity from superior parietal cortex (SPC) to V5

**Fig. 1:**
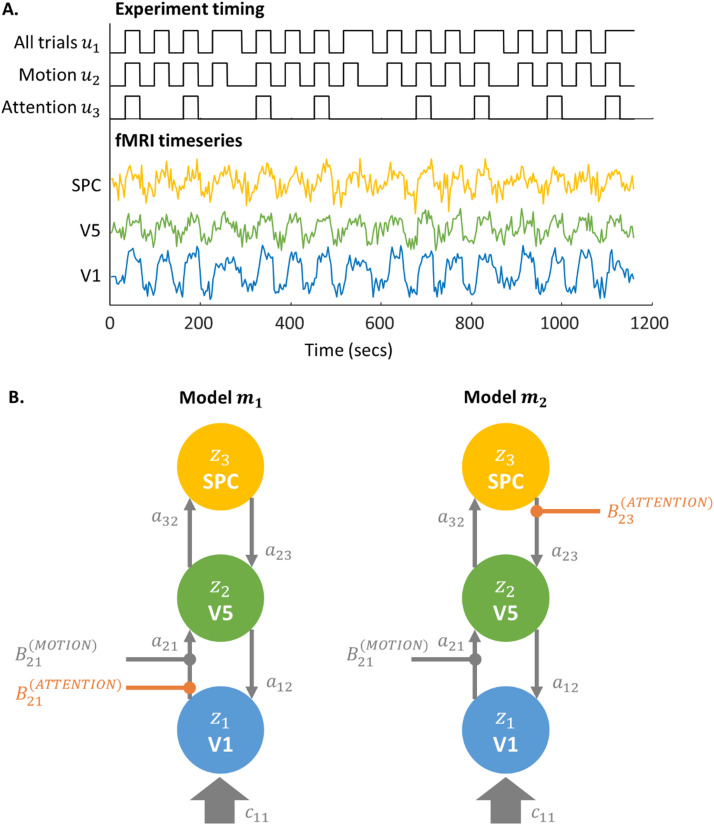
**Exampling modelling problem. A.** Timing of three experimental conditions (top) and representative timeseries fMRI from three brain regions that will be the focus of the analysis (bottom). **B.** Structure of two candidate Dynamic Causal Models (DCMs) used to explain these fMRI data. The models differ only in where Attention has an effect. Each large coloured circle is a brain region, with latent level of neural activity $z_{1},z_{2},z_{3}$ respectively. Arrows between the circles are neural connections encoded in matrix A of the model (see Appendix 1). Arrows with rounded ends encode modulatory effects of Motion and Attention.

Fig. 1A shows the data – fMRI timeseries that were extracted from brain regions V1, V5 and SPC. To formalize the two hypotheses, the next step is to specify models that can generate or simulate the fMRI data that would be expected under each hypothesis, before using the VL scheme to evaluate their evidence.

#### 1.1.1 From hypothesis to models

Models commonly used for fMRI analysis, referred to as Dynamic Causal Models, are specified as follows. Defining a vector $z\left(t\right)=\left(z_{1}\left(t\right),z_{2}\left(t\right),z_{3}\left(t\right)\right)$ to be the overall level of neural activity in V1, V5 and SPC respectively at time t, and vector y(t) to be the (concatenated) fMRI timeseries from all three regions, either hypothesis H1 or H2 can be expressed using the following pair of equations (a state-space model):

(1) $$\begin{array}{ccc} \dot{z}\left(t\right) & = & J\left(t\right)z\left(t\right)+Cu_{1}\left(t\right)\\ y & = & g\left(z,\theta_{h}\right)+\epsilon_{y} \end{array}$$

The first line, which we will call the neural model, describes how the rate of change in the three brain regions, $\dot{z}$, depends on a time-varying connectivity matrix J(t), which is of dimension [3 × 3], and vector of external driving inputs $Cu_{1}\left(t\right)$ . The second line of Eq. (1) includes an *observation model*, g, which translates from neural activity z to fMRI data y. This part of the model is governed by a vector of parameters $\theta_{h}$, which, for MRI, includes the rate of blood flow through the venous compartment. This model, together with the Bayesian methods set out here, are together referred to as DCM for fMRI (Friston et al., 2003).

The parameterisation of J(t) determines which connections are switched on (informed by the data) and which are switched off (fixed at zero). We can therefore formalize our hypotheses by specifying two variants of the model, $m_{1}$ and $m_{2}$, that differ in which connections can be modulated by attention, as illustrated in Fig. 1B. The corresponding parametrisation of J(t) for the two models is provided in Appendix 1, although this is not required for understanding the rest of this article.

Given these two hypotheses, H1 and H2, which we have now formally stated as mathematical models $m_{1}$ and $m_{2}$, we have two overall aims:

1 To estimate the models’ parameters, which includes the strength of neural connections and the effects of each experimental condition on each connection.
2 To estimate the probability for each model given the data, $P(m_{1}|y)$ and $P(m_{2}|y)$, enabling us to select the best model or models (referred to as Bayesian model selection). This requires the intermediate step of estimating the *model evidence*, also called the *marginal likelihood*, which is the probability of having seen the data under each model, $P(y|m_{1})$ and $P(y|m_{2})$.

Both objectives are fulfilled by the VL scheme, which we will introduce next.

### 1.2 VL in a nutshell

To explain the problem that VL solves from a statistical perspective, we begin with Bayes rule. For a model with a vector of parameters θ, we start by defining a *prior* probability density P(θ). This function defines our belief about any particular value of the parameters, or range of values, before seeing the data. The priors serve to regularize or constrain the estimation of the model, making ill-posed problems tractable – in the sense there is a unique solution. We also define a likelihood P(y|θ), which is a function of the parameters θ and returns the probability of observing the data y given those parameters. Together, the priors and likelihood form a *generative model* of the data. The goal of Bayesian inference, as introduced above, is two-fold. First, to obtain an updated probability density over the parameters after seeing the data – the posterior P(θ|y). Second, to obtain the *marginal likelihood* or *model evidence* P(y), which scores the quality of the model and enables models to be compared. If the data ‘look like’ the kind of data that the model would have predicted, then P(y) will be large, whereas if the data are inconsistent with the model, then P(y) will be smaller, and thus models can be compared on the basis of their evidence. This is called *Bayesian model comparison* and we will return later to why the model evidence is well-suited to comparing models. The posterior and evidence are related by Bayes rule:

(2) $$P\left(\theta |y\right)=\frac{P\left(y|\theta \right)p\left(\theta \right)}{P\left(y\right)}=\frac{P\left(y,\theta \right)}{P\left(y\right)}$$

Where the model evidence is the integral or sum over possible settings of the parameters:

(3) P(y)=∫P(y,θ)dθ

Thus, calculating the model evidence involves marginalising (summing or integrating) over the parameters. Unfortunately, this integral typically lacks an analytic solution and cannot be calculated directly. This is problematic for the calculating model evidence, but also the posterior probability (which requires the evidence).

It is the intractability of the integral in Eq. (3) that necessities approximate Bayesian inference. Traditional sampling methods are a common approach for approximating the posterior, which eschew the need to tackle this integral, however they can be very computationally intensive and do not provide a straight-forward way to approximate the evidence, thereby precluding Bayesian model comparison. Instead, the approach described here is to construct a quantity that lower bounds the log of the evidence (i.e., is always smaller than or equal to it). This is often referred to as an evidence lower bound (ELBO) or a free energy functional.^2^ An algorithm is then derived to maximise this bound – making it as close as possible to the log evidence. Methods that involve this optimisation of a bound – and therefore ensure that the ELBO or free energy approximates the log evidence – are collectively referred to as ‘Variational Bayes’ (VB). These methods were first introduced by Richard Feynman in statistical physics (Feynman, 1972) and terms such as ‘free energy’ were retained when it was subsequently applied in different fields. Variational methods were introduced into machine learning though ensemble learning (Hinton and Van Camp, 1993; MacKay, 1995b; MacKay, 1995a). Later, schemes like expectation maximisation (EM) were considered in the light of VB (Bishop, 1998; Neal and Hinton, 1998; Beal, 2003), which proved particularly useful for fitting graphical models (Jordan et al., 1999).

A key practical challenge for the routine use of VB is deriving the necessary model-fitting algorithm for a given model. This is time-consuming and requires a certain degree of skill with variational calculus. Approaches have therefore been developed over the years for implementing VB for a sufficiently broad class of models that new models can be introduced and fitted to data using ‘plug-and-play’ software routines. VL is one such approach (Friston et al., 2007), which has been employed in a large body of work in neuroscience. The reference implementation of VL is a MATLAB function, spm_nlsi_gn,^3^ implemented in the Statistical Parametric Mapping (SPM) software package (https://www.fil.ion.ucl.ac.uk/spm/).

We proceed by deriving a score for the quality of a model: the free energy bound on the log evidence. Then, we derive the algorithm that optimises this bound, providing estimates of the log evidence and posterior over parameters. We illustrate this with worked examples, including the attention to visual motion example above, code for which is provided with this paper. Finally, in the discussion, we consider some advantages and disadvantages of this scheme and its relation to other variational inference software tools currently in use. An outline of mathematical notation appears in the footnote.^4^

## 2 The generative model

The VL scheme works with models that can be written generically in the following form. We have a vector of observed data y of length D and a model g(β), where β are the model parameters:

(4) $$y=g\left(\beta \right)+\epsilon_{y}$$

In the example above, y are fMRI timeseries data and g is a model of how the data were generated, however VL is generic and any data or model could be used. The vector of errors or residuals $\epsilon_{y}$ has a multivariate normal density, with mean zero, and precision matrix $\;\Pi_{y}$ of dimension D (which is the inverse of the covariance matrix):

(5) $$\epsilon_{y}∼\;N\left(0,\Pi_{y}^{-1}\right)$$

Elements on the leading diagonal of matrix $\Pi_{y}$ are the precision (inverse variance) of each measurement, and any non-zero off-diagonal elements encode the conditional dependencies amongst measurements, which determines their correlation. To parameterize $\Pi_{y}$ – such that it can be estimated from the data – we decompose it into a weighted mixture of K matrices called *precision components*, $Q_{k=1,\ldots ,K}\in R_{D\times D}$, each of which has a corresponding scalar hyperparameter $\lambda =(\lambda_{1},\ldots \lambda_{k})$:

(6) $$\Pi_{y}\left(\lambda \right)=\exp\left(\lambda_{1}\right)Q_{1}+\ldots +\exp\left(\lambda_{k}\right)Q_{k}$$

The exponential of each hyperparameter is taken to enforce positivity (because precisions and variances cannot be negative). This approach provides a convenient method for allowing different mixtures of observations to share variance. At its simplest, a single precision component can be used, set to the identity matrix, $Q_{1}=I_{D}$, in which case the corresponding hyperparameter $\lambda_{1}$ encodes the log precision of the observation noise.

To make this into a statistical model, we define a likelihood function P(y|θ) and a prior probability density P(θ), where the parameters and hyperparameters are θ=(β,λ). As set out in the previous section, the likelihood P(y|θ) returns the probability of observing the data y given a particular setting of the parameters θ. This has a multivariate normal density:

(7) $$P\left(y|\beta ,\lambda \right)=N\left(g\left(\beta \right),\Pi_{y}{\left(\lambda \right)}^{-1}\right)$$

Eq. (7) states that the observations are expected to be centred on the prediction of the model g(β), with the level of observation noise given by the precision matrix $\Pi_{y}\left(\lambda \right)$. Next, the prior density P(θ) quantifies our belief about any given value of the parameters before performing the analysis. Here, we define multivariate normal densities as priors over the parameters and hyperparameters:

(8) $$\begin{array}{ccc} P\left(\beta \right) & = & N\left(\eta_{\beta },\Pi_{\beta }^{-1}\right)\\ P\left(\lambda \right) & = & N\left(\eta_{\lambda },\Pi_{\lambda }^{-1}\right) \end{array}$$

Having defined the model, we next set out methods for approximating the log of the model evidence lnP(y) and the posterior P(θ|y).

## 3 Variational Bayes

We will convert the difficult problem of calculating the integral in Eq. (3) into a simpler optimization or search problem. This begins by defining a functional called the free energy, which is a *lower bound* on the log of the model evidence lnP(y). This means that by construction, it can only return values that are less than or equal to the log evidence. Then we'll search for a setting of the parameters of the free energy (*variational parameters*) that maximize it, making it as close as possible to the unknown log evidence. Helpfully, the parameters that maximize the bound will turn out to approximate the posterior P(θ|y).

### 3.1 Constructing a lower bound on the log evidence

The log evidence is defined as:

(9) lnP(y)=ln∫P(y,θ)dθ

**Fig. 2:**
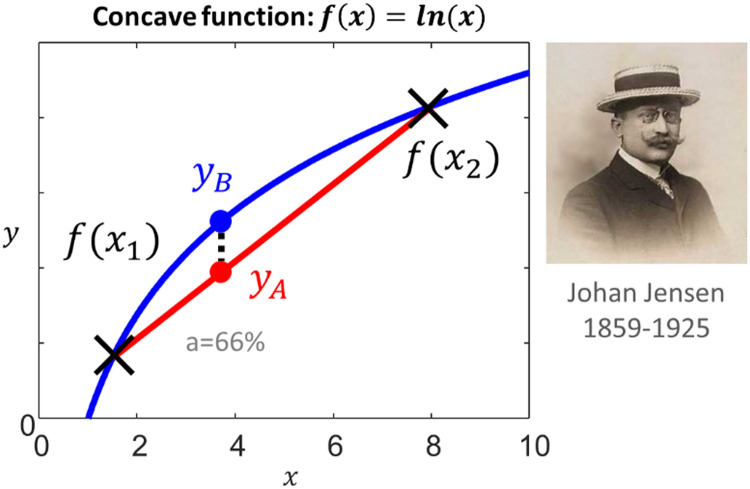
**Jensen's inequality**. The curved blue line could be any concave function y=f(x). Here as an example, the function f(x)=ln(x) is shown, which has been evaluated in the range x=[0,10]. The straight red *secant line* joints two arbitrary points: $f(x_{1})$ and $f(x_{2})$. Any point on the secant line, such as $y_{A}$, is a weighted average of $f(x_{1})$ and $f(x_{2})$: $y_{A}=af\left(x_{1}\right)+\left(1-a\right)f\left(x_{2}\right)$. Jensen's inequality says that this average-of-functions will always be less than or equal to the corresponding function-of-the-average: $y_{B}=f\left(ax_{1}+\left(1-a\right)x_{2}\right)$. Thus, using the notation of expected values, E[f(x)]≤f(E[X]). Portrait in the public domain via *Wikipedia*. https://en.wikipedia.org/wiki/Johan_Jensen_(mathematician).

To construct a lower bound on this quantity, we will make use of Jensen's inequality, which says that the *average of a log* is always less than or equal to the *log of an average* (Fig. 2). That means that if we can express lnP(y) as the average of a log, then to construct a lower bound we simply need to rearrange terms to get the log of an average.

To express the log evidence as the average of a log, we first define a probability density Q(θ) that will form our approximation of the posterior over parameters. We then introduce this density into the log evidence (from Eq. (9)), multiplying and dividing so that it causes no overall change to the evidence:

(10) $$\begin{array}{ccc} \ln\;P\left(y\right) & = & \ln\int \frac{Q\left(\theta \right)}{Q\left(\theta \right)}P\left(y,\theta \right)\;d\theta \\ & = & \text{ln}\;E_{Q\left(\theta \right)}\left[\frac{P\left(y,\theta \right)}{Q\left(\theta \right)}\right] \end{array}$$

Where E[·] is the expected value or average.^5^ This is the log of an average, so (by Jensen's inequality) a lower bound on this will be the average of the log, which is called the free energy F:

(11) $$F\left[Q\left(\theta \right)\right]=E_{Q\left(\theta \right)}\left[\ln\frac{P\left(y,\theta \right)}{Q\left(\theta \right)}\right]$$

Here, we have written the free energy as a functional (a function of a function), with Q(θ) as its input. By construction, for any choice of probability density Q(θ), it holds that F[Q(θ)]≤lnP(y). Note that the free energy is sometimes defined to be the negative of this quantity. We have used the terminology typically found in the neuroimaging literature, where the free energy is a lower bound on the log evidence, also known as the ELBO in statistical and machine learning. The next step will be to maximize this lower bound, i.e., find the probability density Q(θ) that maximizes the free energy, bringing it close to the log evidence, so F≈lnP(y).

### 3.2 Useful properties of the free energy

The free energy can be rewritten in different ways to illustrate why it serves as a useful score for the quality of a model. First, we can rewrite it as the log evidence minus the Kullback-Leibler (KL) divergence between the true and approximate posterior:

(12) $$\begin{array}{ccc} F\left[Q\left(\theta \right)\right] & = & E_{Q\left(\theta \right)}\left[\ln\frac{P\left(\theta |y\right)P\left(y\right)}{Q\left(\theta \right)}\right]\\ & = & E_{Q\left(\theta \right)}\left[\ln\;P\left(\theta |y\right)+\ln\;P\left(y\right)-\ln\;Q\left(\theta \right)\right]\\ & = & \ln\;P\left(y\right)+E_{Q\left(\theta \right)}\left[\ln\frac{P\left(\theta |y\right)}{Q\left(\theta \right)}\right]\\ & = & \underset{\text{Log}\;\text{evidence}}{\underset{‾}{\ln\;P\left(y\right)}}-\underset{\text{Approximation}\;\text{error}}{\underset{‾}{D_{KL}\left[Q\left(\theta \right)∥P\left(\theta |y\right)\right]}} \end{array}$$

Where $D_{KL}\left[Q∥P\right]$ is the KL divergence from density P to Q, which is a (non-negative) measure of difference between the two densities. The log evidence is a fixed (but unknown) quantity, so if we can find a density Q(θ) that maximizes the free energy, then we minimize the divergence between the true posterior P(θ|y) and the approximation Q(θ).

We can also re-write the free energy from Eq. (11) as the difference between the model's accuracy and its complexity:

(13) $$\begin{array}{ccc} F\left[Q\left(\theta \right)\right] & = & E_{Q\left(\theta \right)}\left[\ln\;P\left(y|\theta \right)+\ln\;P\left(\theta \right)-\ln\;Q\left(\theta \right)\right]\\ & = & E_{Q\left(\theta \right)}\left[\ln\;P\left(y|\theta \right)+\ln\frac{P\left(\theta \right)}{Q\left(\theta \right)}\right]\\ & = & \underset{\text{Accuracy}}{\underset{‾}{E_{Q\left(\theta \right)}\left[\ln\;P\left(y|\theta \right)\right]}}-\underset{\text{Complexity}}{\underset{‾}{D_{KL}\left[Q\left(\theta \right)∥P\left(\theta \right)\right]}} \end{array}$$

Here, the accuracy term is the expected log likelihood, and the complexity is how far the parameters have diverged from the prior to the approximate posterior. Thus, if we select the model with the most positive free energy out of several candidate models, then we inherently select the model that offers the best trade-off between accuracy and complexity (c.f., Occam's razor). Importantly, this definition of complexity takes into account the covariance amongst parameters, and thus enables the free energy to serve as a better approximation of the log evidence than statistics which discard the covariance – in particular the BIC and AIC (Penny, 2012b).

Finally, we can re-arrange the free energy in Eq. (11) as follows:

(14) $$\begin{array}{ccc} F\left[Q\left(\theta \right)\right] & = & E_{Q\left(\theta \right)}\left[\ln\;P\left(y,\theta \right)\right]-E_{Q\left(\theta \right)}\left[\ln\;Q\left(\theta \right)\right]\\ & = & \underset{\text{Expected}\;(\text{negative})\;\text{energy}}{\underset{‾}{E_{Q\left(\theta \right)}\left[\ln\;P\left(y,\theta \right)\right]}}+\underset{\text{Entropy}}{\underset{‾}{H\left[Q\left(\theta \right)\right]}} \end{array}$$

By analogy with its applications in statistical physics, the first term is referred to as an *energy*, and the latter is the Shannon entropy H of the posterior density Q(θ). A probability density Q(θ) with high entropy will be smooth (or in the limit, flat) over the possible values of θ, whereas a probability density with low entropy will have peaks around particular values. This means that if we have two candidate Q(θ) densities, both equally likely under the priors, to maximize the free energy we would select the one that is smoother or, equivalently, with the higher entropy. This is referred to as Jaynes’ Principle of Maximum Entropy (Jaynes, 1957), and means that we select the simplest explanation for the data where possible. (A further consequence of Jaynes’ Principle is that as we come closer to maximizing the free energy, the free energy forms a smoother landscape, aiding the performance of optimization algorithms. We will return to the notion of a free energy landscape in Section 5).

## 4 Free energy under the Laplace approximation

We now build up to the definition of the free energy that is used in the VL scheme, by first defining the probability densities that appear in the definition of the free energy (Eq. (11)) and then by introducing a *mean-field partition* over parameters and hyperparameters.

### 4.1 Quadratic approximations

In what follows, we will approximate the various unknown probability densities that appear in Eq. (11) using multivariate normal densities, by way of *quadratic approximations* and *Laplace's method*, which we will first reprise. A quadratic approximation, also called a second order Taylor approximation, approximates any (twice differentiable) function g(x) that has vector input x, close to its peak or mode $x=x_{0}$, with the linear function:

(15) $$T\left(x\right)=\underset{\text{Constant}}{\underset{‾}{g\left(x_{0}\right)}}+\underset{\text{Linear}\;\text{term}=0}{\underset{‾}{\nabla g\left(x_{0}\right)\cdot \left(x-x_{0}\right)}}+\underset{\text{Quadratic}\;\text{term}}{\underset{‾}{\frac{1}{2}{\left(x-x_{0}\right)}^{T}H_{g}\left(x_{0}\right)\left(x-x_{0}\right)}}$$

(16) $$=\underset{\text{Constant}}{\underset{‾}{g\left(x_{0}\right)}}+\underset{\text{Quadratic}\;\text{term}}{\underset{‾}{\frac{1}{2}{\left(x-x_{0}\right)}^{T}H_{g}\left(x_{0}\right)\left(x-x_{0}\right)}}$$

where $\nabla g(x_{0})$ is the gradient of function g evaluated at $x_{0}$ and $H_{g}\left(x_{0}\right)$ is the Hessian matrix—a matrix of second derivatives—of g evaluated at $x_{0}$, i.e., ${\left[H_{g}\left(x_{0}\right)\right]}_{i,j}=\partial_{x_{i}x_{j}}g\left(x_{0}\right)$. At the mode of the density, $x=x_{0}$, the linear term equals zero and thus disappears in Eq. (15). This has a very similar form to the log of a normal density, which is utilized in *Laplace's method* for function approximation.

### 4.2 Laplace's method

**Fig. 3:**
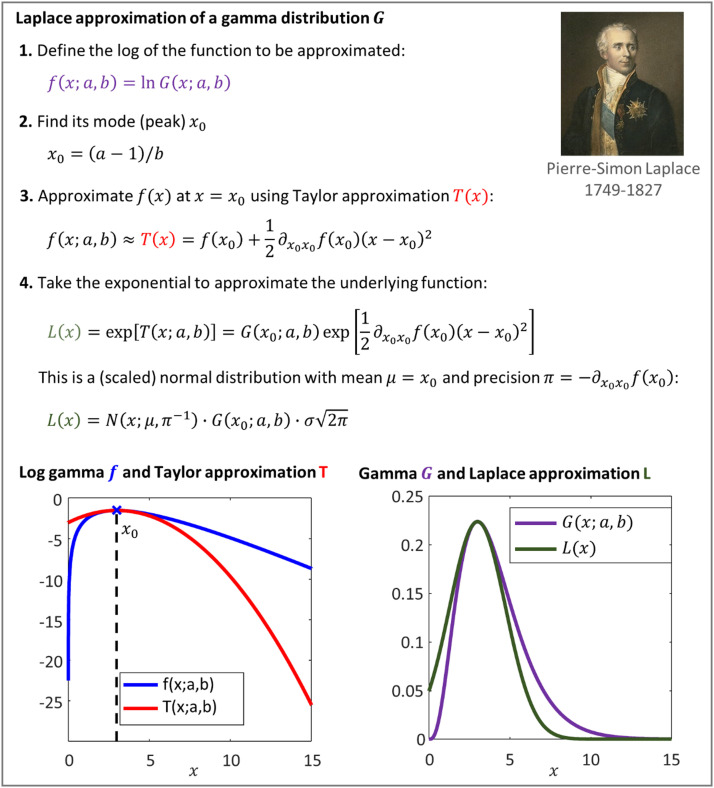
**Laplace approximation of a non-normal density**. This example illustrates Laplace's method by approximating a gamma probability density function G(x;a,b) over the variable x with shape parameter a and inverse scale parameter b. The result is a scaled normal density $N(x;\mu ,\pi^{-1})$ with mean $\mu =x_{0}$ and precision $\pi =-\partial_{x_{0}x_{0}}\ln G\left(x_{0}\right)$, where $x_{0}$ is the mode of lnG. After normalisation to ensure it is a proper probability density, the scaling becomes irrelevant, and we end up with a normal density. Portrait by James Posselwhite, in the public domain via *Wikipedia*, https://en.wikipedia.org/wiki/Pierre-Simon_Laplace.

Laplace's method leverages the quadratic approximation in order to approximate a function g near its mode as a (scaled) normal density, as detailed in Fig. 3. If g(x) is a function of scalar variable x, the Laplace approximation L(x) is:

(17) $$\begin{array}{ccc} g\left(x\right) & \approx & L\left(x\right)\propto N\left(x;\mu ,\pi^{-1}\right)\\ \mu & = & x_{0}\\ \pi & = & -\partial_{x_{0}x_{0}}\ln\;g\left(x_{0}\right) \end{array}$$

where μ is the mean and π is the precision. When g(x) is a function of a vector of variables x, the Laplace approximation L(x) returns a (scaled) multivariate normal density:

(18) $$\begin{array}{ccc} g\left(x\right) & \approx & L\left(x\right)\propto N\left(x;\mu ,\Pi^{-1}\right)\\ \mu & = & x_{0}\\ \Pi & = & -\partial_{x_{0}x_{0}}\ln\;g\left(x_{0}\right) \end{array}$$

Where μ is the mean and Π is the precision matrix, i.e., the inverse of the variance-covariance matrix. We will apply this method several times to approximate the different quantities that comprise the free energy.

### 4.3 Approximating the free energy

Recall Eq. (11), the definition of the free energy: $F\left[Q(\theta )\right]=E_{Q(\theta )}\left[\ln\;P(y,\theta )-\ln\;Q(\theta )\right]$. We will now write expressions for the log joint lnP(y,θ) and the approximate posterior (i.e., the choice of Q(θ)). As the true form of the posterior is unknown, and the form of the log joint may involve analytically difficult nonlinearities, we will use (local) quadratic or Laplace approximations to define them in a flexible manner.

#### 4.3.1 Approximating lnP(y,θ)

We will apply a quadratic approximation for the log joint density, which is reasonable because densities tend to look normal close to their mode. We define μ to be the mode of the joint probability density, $\mu =\underset{\theta }{\text{argmax}}P\left(y,\theta \right)$. Note that this is the same as the mode of the posterior P(θ|y), because the posterior is simply a scaled version of the joint. (Also, μ is the mode of the log of these densities, as taking the logarithm does not change the mode.) The approximation to the log joint is:

(19) $$\begin{array}{ccc} \ln\;P\left(y,\theta \right) & \approx & \ln\;P\left(y,\mu \right)+\frac{1}{2}{\left(\theta -\mu \right)}^{T}\left[\partial_{\mu \mu }\ln\;P\left(y,\mu \right)\right]\left(\theta -\mu \right)\\ & = & \ln\;P\left(y,\mu \right)-\frac{1}{2}{\left(\theta -\mu \right)}^{T}\Sigma^{-1}\left(\theta -\mu \right)\\ \Sigma^{-1} & = & -\partial_{\mu \mu }\ln\;P\left(y,\mu \right)=-\partial_{\mu \mu }\ln\;P\left(\mu |y\right) \end{array}$$

#### 4.3.2 Choosing an approximate posterior Q

We will similarly apply a quadratic approximation to the log posterior lnP(θ|y) around the posterior mode μ. Under Laplace's method, this means we will be using a normal density, Q(θ), in order to approximate the posterior P(θ|y):

(20) $$\begin{array}{ccc} \ln\;P\left(\theta |y\right) & \approx & \ln\;P\left(\mu |y\right)-\frac{1}{2}{\left(\theta -\mu \right)}^{T}\Sigma^{-1}\left(\theta -\mu \right)\\ \Rightarrow P\left(\theta |y\right) & \approx & Q\left(\theta \right)=N\left(\mu ,\Sigma \right) \end{array}$$

#### 4.3.3 Taking expectations

Next, we will apply the expectation operator^6^ from Eq. (11) to the previous two densities under the approximate posterior:

(21) $$E_{Q\left(\theta \right)}\left[\ln\;P\left(y,\theta \right)\right]\approx \ln\;P\left(y,\mu \right)-\frac{1}{2}E_{Q\left(\theta \right)}\left[{\left(\theta -\mu \right)}^{T}\Sigma^{-1}\left(\theta -\mu \right)\right]$$

(22) $$E_{Q\left(\theta \right)}\left[\ln\;Q\left(\theta \right)\right]\approx -\frac{1}{2}\left[\ln\;\left(\left|\Sigma \right|\right)+n\ln\;2\pi \right]-\frac{1}{2}E_{Q\left(\theta \right)}\left[{\left(\theta -\mu \right)}^{T}\Sigma^{-1}\left(\theta -\mu \right)\right]$$

Where Eq. (22) uses the definition of the log of the multivariate normal density for a variable with dimension n.

To simplify these expressions, note that each quadratic term inside the square brackets is a scalar. This means we can use the ‘trace trick’, tr(ABC)=tr(CAB). Applying this gives the simpler expressions:

(23) $$\begin{array}{ccc} E_{Q\left(\theta \right)}\left[\ln\;P\left(y,\theta \right)\right] & \approx & \ln\;P\left(y,\mu \right)-\frac{1}{2}E_{Q\left(\theta \right)}\left[tr\left(\underset{A}{\underset{‾}{{\left(\theta -\mu \right)}^{T}}}\underset{B}{\underset{‾}{\Sigma^{-1}}}\underset{C}{\underset{‾}{\left(\theta -\mu \right)}}\right)\right]\\ & = & \ln\;P\left(y,\mu \right)-\frac{1}{2}tr\left(E_{Q\left(\theta \right)}\left[\underset{C}{\underset{‾}{\left(\theta -\mu \right)}}\underset{A}{\underset{‾}{{\left(\theta -\mu \right)}^{T}}}\right]\underset{B}{\underset{‾}{\Sigma^{-1}}}\right)\\ & = & \ln\;P\left(y,\mu \right)-\frac{1}{2}tr\left(\Sigma \;\Sigma^{-1}\right)\\ & = & \ln\;P\left(y,\mu \right)-\frac{n}{2} \end{array}$$

(24) $$\begin{array}{ccc} E_{Q\left(\theta \right)}\left[\ln\;Q\left(\theta \right)\right] & = & -\frac{1}{2}\left[\ln\;\left(\left|\Sigma \right|\right)+n\ln\;2\pi \right]-\frac{1}{2}E_{Q\left(\theta \right)}\left[\underset{A}{\underset{‾}{{\left(\theta -\mu \right)}^{T}}}\underset{B}{\underset{‾}{\Sigma^{-1}}}\underset{C}{\underset{‾}{\left(\theta -\mu \right)}}\right]\\ & = & -\frac{1}{2}\left[\ln\;\left(\left|\Sigma \right|\right)+n\ln\;2\pi \right]-\frac{1}{2}tr\left(E_{Q\left(\theta \right)}\left[\underset{C}{\underset{‾}{\left(\theta -\mu \right)}}\underset{A}{\underset{‾}{{\left(\theta -\mu \right)}^{T}}}\right]\underset{B}{\underset{‾}{\Sigma^{-1}}}\right)\\ & = & -\frac{1}{2}\left[\ln\left(\left|\Sigma \right|\right)+n\ln\;2\pi \right]-\frac{1}{2}tr\left(\Sigma \Sigma^{-1}\right)\\ & = & -\frac{1}{2}\left[\ln\left(\left|\Sigma \right|\right)+n\ln\;2\pi \right]-\frac{n}{2}\\ & = & -\frac{1}{2}\left[\ln\left(\left|\Sigma \right|\right)+n\ln\;2\pi e\right] \end{array}$$

Substituting these expectations into Eq. (11), we get the free energy under the Laplace approximation:

(25) $$\begin{array}{ccc} F\left[Q\left(\theta \right)\right] & = & E_{Q\left(\theta \right)}\left[\ln\;P\left(y,\theta \right)-\ln\;Q\left(\theta \right)\right]\\ & = & \ln\;P\left(y,\mu \right)-\frac{n}{2}+\frac{1}{2}\left[\ln\left(\left|\Sigma \right|\right)+n\ln\;2\pi e\right] \end{array}$$

Where Σ is the posterior covariance and n is the total number of parameters.

### 4.4 Free energy under a mean-field approximation

The previous section assumed that all parameters are treated equally. However, it is often helpful to separate them out into two (or more) types. The estimation scheme outlined here alternates between updating the estimate of the model's parameters β and the hyperparameters λ that control the precision of the observation noise. We previously lumped these two kinds of parameter together as θ but now separate them out. We can re-write the joint probability as follows:

(26) P(y,β,λ)=P(y|β,λ)P(β)P(λ)

with normal densities for the likelihood and priors Eqs. (7) and (8). The approximate posterior is chosen to factorise as follows:

(27) Q(θ)=Q(β,λ)=Q(β)Q(λ)

This factorisation is known as a mean-field approximation. Each approximate posterior is a multivariate normal density:

$$Q\left(\beta \right)=N\left(\mu_{\beta },\Sigma_{\beta }\right)$$

(28) $$Q\left(\lambda \right)=N\left(\mu_{\lambda },\Sigma_{\lambda }\right)$$

The free energy is easily extended for this factorisation (from Eq. (25)):

(29) $$\begin{array}{ccc} F\left[Q\left(\theta \right)\right] & = & \underset{E_{Q\left(\theta \right)}\left[\ln\;P\left(y,\theta \right)\right]}{\underset{‾}{\ln\;P\left(y,\mu_{\beta },\mu_{\lambda }\right)-\frac{1}{2}\left(p+h\right)}}\\ & & +\underset{E_{Q\left(\theta \right)}\left[\ln\;Q\left(\theta \right)\right]}{\underset{‾}{\frac{1}{2}\left[\ln\left(\left|\Sigma_{\beta }\right|\right)+\ln\left(\left|\Sigma_{\lambda }\right|\right)+\left(p+h\right)\ln\;2\pi e\right]}}\\ & = & \ln\;P\left(y,\mu_{\beta },\mu_{\lambda }\right)+\frac{1}{2}\left[\ln\left(\left|\Sigma_{\beta }\right|\right)+\ln\left(\left|\Sigma_{\lambda }\right|\right)+\left(p+h\right)\ln\;2\pi \right] \end{array}$$

Where p is the number of parameters and h is the number of hyperparameters.

So, we now just need to define the log joint, which is the product of three normal densities in Eq. (26). The log of a normal density with mean m covariance matrix S for a variable of dimension k is defined as:

(30) $$\begin{array}{ccc} \ln\;N\left(x;m,S\right) & = & -\frac{1}{2}\left[\ln\left|S\right|+{\left(x-m\right)}^{T}S^{-1}\left(x-m\right)+k\ln\left(2\pi \right)\right]\\ & = & -\frac{1}{2}\left[\ln\left|S\right|+\epsilon_{x}^{T}S^{-1}\epsilon_{x}+k\ln\left(2\pi \right)\right] \end{array}$$

Where the *error term* is $\epsilon_{x}=x-\mu$. Substituting into Eq. (29) (with dim(y)=k):

(31) $$\begin{array}{ccc} F\left[Q\left(\theta \right)\right] & = & \ln\;P\left(y|\beta ,\;\lambda \right)+\ln\;P\left(\beta \right)+\ln\;P\left(\lambda \right)+\frac{1}{2}\left[\ln\left(\left|\Sigma_{\beta }\right|\right)+\ln\left(\left|\Sigma_{\lambda }\right|\right)+\left(p+h\right)\ln\;2\pi \right]\\ & = & \underset{\text{Likelihood}}{\underset{‾}{-\frac{1}{2}\left[\ln\left|\Pi_{y}^{-1}\right|+\epsilon_{y}^{T}\Pi_{y}\epsilon_{y}\right]}}\\ & & \underset{\text{Prior}\;(\text{parameters})}{\underset{‾}{-\frac{1}{2}\left[\ln\left|\Pi_{\beta }^{-1}\right|+\epsilon_{\beta }^{T}\Pi_{\beta }\epsilon_{\beta }\right]}}\\ & & \underset{\text{Prior}\;(\text{hyperparameters})}{\underset{‾}{-\frac{1}{2}\left[\ln\left|\Pi_{\lambda }^{-1}\right|+\epsilon_{\lambda }^{T}\Pi_{\lambda }\epsilon_{\lambda }\right]}}\\ & & \underset{\text{Posterior}\;\text{entropy}}{\underset{‾}{+\frac{1}{2}\left[\ln\left(\left|\Sigma_{\beta }\right|\right)+\ln\left(\left|\Sigma_{\lambda }\right|\right)\right]}}\\ & & \underset{\text{Constants}}{\underset{‾}{-\frac{1}{2}k\ln\left(2\pi \right)}} \end{array}$$

With error terms:

$$\epsilon_{y}=y-g\left(\mu_{\beta }\right)$$

$$\epsilon_{\beta }=\mu_{\beta }-\eta_{\beta }$$

(32) $$\epsilon_{\lambda }=\mu_{\lambda }-\eta_{\lambda }$$

Where, to recap, $\Pi_{\beta },\;\Pi_{\lambda }$ are prior precisions, $\eta_{\beta }$, $\eta_{\lambda }$ are prior expectations, $\Sigma_{\beta },\Sigma_{\lambda }$ are posterior covariances, $\mu_{\beta }$,$\;\mu_{\lambda }$ are posterior expectations and $\Pi_{y}$ is the (modelled) precision of the data. We then use Eq. (31) as the objective function (i.e., the measure of the model's quality), which we seek to maximize in order to approximate the log evidence and identify the model's parameters.

## 5 Estimation scheme

Next, we derive an algorithm for finding the posterior parameter density Q(θ) that maximizes the free energy F[Q(θ)]. Full derivations of these equations are provided in the supplementary material.

### 5.1 Overview of gradient ascent and Gauss-Newton

**Fig. 4:**
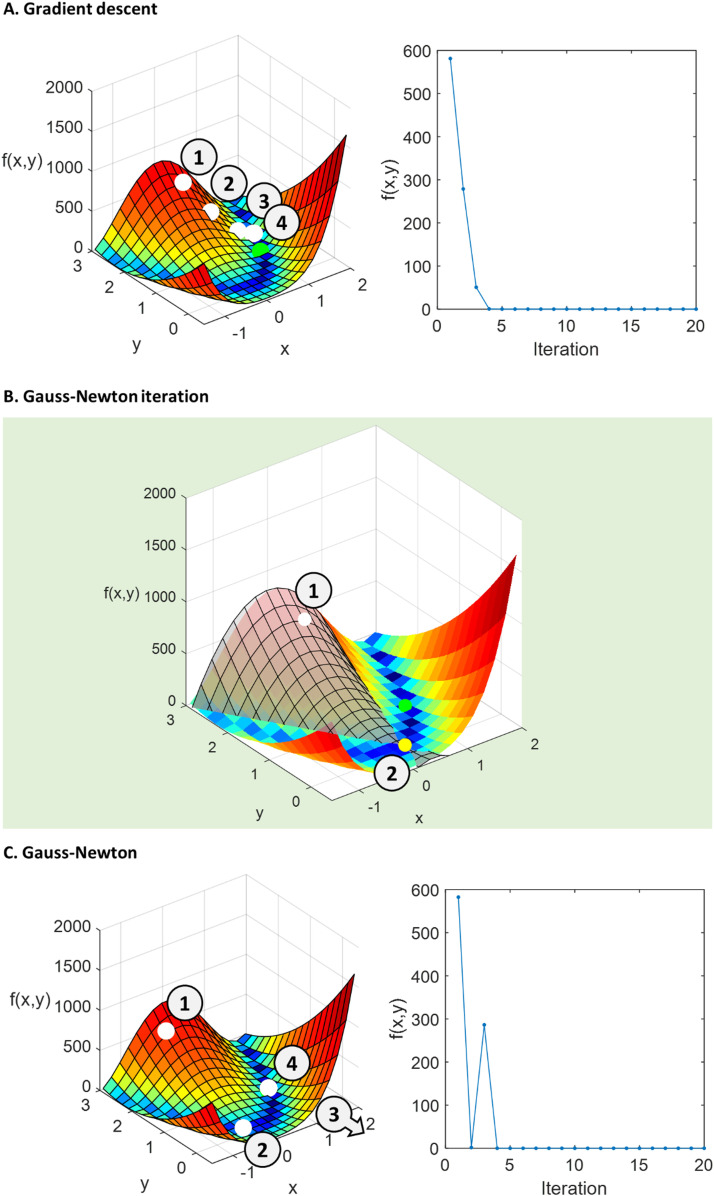
**Comparison of Gradient ascent and Gauss-Newton optimization**. The left and centre panels illustrate an example function with two inputs f(x,y) (the Rosenbrock function, with hyperparameters *a* = 1, *b* = 100). The global minimum is at *x* = 1, *y* = 1, indicated with a shaded green sphere in panel A. White spheres are estimates of the parameters (x,y) over successive iterations, indexed by the numbers in circles. The right panels show the evaluation of the function (i.e., the height of the surface) over successive iterations.

The simplest approach to numerically maximizing the free energy is *gradient ascent*. Conceptually, the free energy forms a landscape, the dimensions of which are the parameters. Gradient ascent or descent takes small steps in the same direction as the gradient, as if climbing or descending a hill. Writing the function to be optimized generically as f(μ), the parameters μ are updated on each iteration according to μ =μ+Δμ**,** where:

(33) $$\Delta \mu =\alpha \nabla_{\mu }\left[f\left(\mu \right)\right]$$

and α is the step size (positive for ascent, and negative for descent). This is illustrated in Fig. 4A, for finding the minimum of a function using gradient descent. From an initial guess (labelled 1), small steps are taken towards a minimum, which in this example happens to be the global minimum (shaded green sphere).

Gradient ascent or descent is rarely sufficient in practice. One issue regards its speed: because the update is proportional to the gradient, estimates advance very slowly in shallow regions of the landscape (as can be seen in Fig. 4A). Conversely, in very steep regions, the estimates advance quickly – running the risk of taking too large a step and moving away from an optimum. We would ideally like the opposite situation – to move quickly in shallow regions, and slowly in steeper regions.

The Gauss-Newton algorithm takes a different approach and can converge far more quickly. To minimize a function, a parabola (or U-shaped plane) is fitted to f(μ) at the current estimate of the parameters (Fig. 4B). The algorithm then jumps to the minimum of the parabola, which becomes the new parameter estimate (labelled 2 in Fig. 4B) and the process repeats. This can enable fast progression from the initial estimate to the global or local optimum (Fig. 4C). For some vector of parameters $\mu^{*}$, the parabola is a second-order quadratic approximation of f(μ) at the current estimate of the parameters μ:

(34) $$f\left(\mu^{*}\right)\approx f\left(\mu \right)+\nabla_{\mu }f\left(\mu \right)\cdot \left(\mu^{*}-\mu \right)+\frac{1}{2}\left(\mu^{*}-\mu \right)H_{f}\left(\mu \right)\left(\mu^{*}-\mu \right)$$

Where $H_{f}$ is the Hessian matrix of second derivatives. This approximation has a unique minimum at $\mu -H_{f}{\left(\mu \right)}^{-1}\cdot \nabla_{\mu }f\left(\mu \right)$, which becomes the new estimate of the parameters, giving rise to the update equation:

(35) $$\Delta \mu =-H_{f}{\left(\mu \right)}^{-1}\cdot \nabla_{\mu }\left[f\left(\mu \right)\right]$$

While Gauss-Newton is much faster than gradient ascent, it will perform poorly if the quadratic approximation is poor – a particular risk when taking large steps that are far from the current estimate. In these situations, it would be ideal to dynamically switch to a gradient ascent or descent. A continuous transition between gradient ascent and Gauss-Newton like behaviour is achieved by the variational Laplace scheme, as we will return to shortly. As a first step towards this, we will derive a gradient ascent algorithm for the free energy.

### 5.2 Gradient ascent on free energy

The mean-field approximation Q(θ)=Q(β)Q(λ) naturally gives rise to a gradient ascent algorithm that alternates between optimising the parameters and hyperparameters. With the mean-field approximation, the free energy (Eq. (11)) can be extended to:

(36) $$F\left[Q\left(\theta \right)\right]=E_{Q\left(\beta \right)Q\left(\lambda \right)}\left[\ln\frac{P\left(y,\beta ,\lambda \right)}{Q\left(\beta \right)Q\left(\lambda \right)}\right]$$

We will use $\delta_{Q(\beta )}F$ and $\delta_{Q(\lambda )}F$ to denote the *variation* of the free energy with respect to each factor of the approximate posterior, Q(β) and Q(λ), respectively. These are functional derivatives, encoding the rate of change in F that would result from infinitesimal adjustments to the form of each function—where the functions in question here are probability densities. Ignoring constants:

$$\delta_{Q\left(\beta \right)}F=-\ln\;Q\left(\beta \right)+E_{Q\left(\lambda \right)}\left[\ln\;P\left(y,\beta ,\lambda \right)\right]$$

(37) $$\delta_{Q\left(\lambda \right)}F=-\ln\;Q\left(\lambda \right)+E_{Q\left(\beta \right)}\left[\ln\;P\left(y,\beta ,\lambda \right)\right]$$

Setting to zero and taking the exponential, we get the optimal approximate posteriors on each iteration of the algorithm:

$$\delta_{Q\left(\beta \right)}F=0\Leftrightarrow Q\left(\beta \right)\;\propto \;\exp\left(E_{Q\left(\lambda \right)}\left[\ln\;P\left(y,\beta ,\lambda \right)\right]\right)$$

(38) $$\delta_{Q\left(\lambda \right)}F=0\Leftrightarrow Q\left(\lambda \right)\;\propto \;\exp\left(E_{Q\left(\beta \right)}\left[\ln\;P\left(y,\beta ,\lambda \right)\right]\right)$$

As before (Eq. (23)), quadratic approximations can be used for the terms inside the expectations:

$$E_{Q\left(\beta \right)}\left[\ln\;P\left(y,\beta ,\lambda \right)\right]\approx \ln\;P\left(y,\mu_{\beta },\lambda \right)+\frac{1}{2}tr\left(\Sigma_{\beta }\;\partial_{\mu_{\beta }\mu_{\beta }}\ln\;P\left(y,\mu_{\beta },\lambda \right)\right)$$

(39) $$E_{Q\left(\lambda \right)}\left[\ln\;P\left(y,\beta ,\lambda \right)\right]\approx \ln\;P\left(y,\beta ,\mu_{\lambda }\right)+\frac{1}{2}tr\left(\Sigma_{\lambda }\;\partial_{\mu_{\lambda }\mu_{\lambda }}\ln\;P\left(y,\beta ,\mu_{\lambda }\right)\right)$$

By definition, the modes of the approximate posterior densities over parameters $\mu_{\beta }$ and hyperparameters $\mu_{\lambda }$ must maximise the above quantities (i.e., the logarithms of the approximate posteriors), giving the following pair of equations:

(40) $$\begin{array}{ccc} {\Delta \mu }_{\beta } & = & \nabla_{\mu_{\beta }}E_{Q\left(\lambda \right)}\left[\text{ln}\;P\left(y,\mu_{\beta },\lambda \right)\right]\\ & = & \nabla_{\mu_{\beta }}\text{ln}\;P\left(y,\mu_{\beta },\mu_{\lambda }\right)+\nabla_{\mu_{\beta }}\frac{1}{2}\text{tr}\left(\Sigma_{\lambda }\;\partial_{\mu_{\lambda }\mu_{\lambda }}\text{ln}\;P\left(y,\mu_{\beta },\mu_{\lambda }\right)\right)\\ & = & \underset{\text{Likelihood}}{\underset{‾}{J_{g}^{T}\Pi_{y}\epsilon_{y}}}-\underset{\text{Prior}}{\underset{‾}{\Pi_{\beta }\epsilon_{\beta }}}+\underset{\text{Hyperparameters}\;\lambda }{\underset{‾}{\sum_{j=1}^{h}\left[{\left(\Sigma_{\lambda }\right)}_{\mathrm{jj}}J_{g}^{T}P_{j}\epsilon_{y}\right]}}\\ {\left({\Delta \mu }_{\lambda }\right)}_{i} & = & \partial_{\mu_{\lambda_{i}}}E_{Q\left(\beta \right)}\left[\text{ln}\;P\left(y,\beta ,\mu_{\lambda }\right)\right]\\ & = & \partial_{\mu_{\lambda_{i}}}\text{ln}\;P\left(y,\mu_{\beta },\mu_{\lambda }\right)+\partial_{\mu_{\lambda_{i}}}\frac{1}{2}\text{tr}\left(\Sigma_{\beta }\partial_{\mu_{\beta }\mu_{\beta }}\text{ln}\;P\left(y,\mu_{\beta },\mu_{\lambda }\right)\right)\\ & = & \underset{\text{Likelihood}}{\underset{‾}{\frac{1}{2}\text{tr}\left(P_{i}\Pi_{y}^{-1}\right)-\frac{1}{2}\epsilon_{y}^{T}\;P_{i}\epsilon_{y}}}-\underset{\text{Prior}}{\underset{‾}{\partial_{\mu_{\lambda_{i}}}{\left(\epsilon_{\lambda }\right)}^{T}\Pi_{\lambda }\epsilon_{\lambda }}}-\underset{\text{Parameters}\;\beta }{\underset{‾}{\frac{1}{2}\text{tr}\left(\Sigma_{\beta }J_{g}^{T}\;P_{i}J_{g}\right)}} \end{array}$$

Where vector $\Delta \mu_{\beta }$ is the change in the parameters at each iteration of the algorithm, ${\left(\Delta \mu_{\lambda }\right)}_{i}$ is the change in the i-th hyperparameter at each iteration, $\nabla_{\mu_{\beta }}\left[\cdot \right]$ is the gradient with respect to the parameters and $J_{g}$ is the Jacobian matrix^7^ of first partial derivatives with ${\left(J_{g}\right)}_{ij}=\partial_{\mu_{j}}g_{i}\left(\mu_{\beta }\right)$ for observation i and parameter j. For hyperparameter i, the derivative term $\partial_{\mu_{\lambda_{i}}}\left(\epsilon_{\lambda }\right)$ is a vector with value of unity in index i and zero elsewhere, and $P_{i}=\partial_{\mu_{\lambda_{i}}}\left(\Pi_{y}\right)=\exp\left(\lambda_{i}\right)\Pi_{i}$. Terms including the second derivative of g(θ) have been omitted on the assumption that it is locally approximately linear. Similarly, the final term for the parameters is usually ignored, because its contribution is usually trivial in relation to the other terms, and is zero when the precision is linear in the hyperparameters.

These updates depend on first calculating the covariance of the parameters $\Sigma_{\beta }$ and hyperparameters $\Sigma_{\lambda }$, which are given by the expressions^8^:

(41) $$\begin{array}{ccc} {\left(\Sigma_{\beta }\right)}^{-1} & = & -\partial_{\mu_{\beta }\mu_{\beta }}E_{Q\left(\lambda \right)}\left[\ln\;P\left(y,\mu_{\beta },\lambda \right)\right]\\ & \approx & J_{g}^{T}\Pi_{y}J_{g}+\Pi_{\beta }\\ {\left[{\left(\Sigma_{\lambda }\right)}^{-1}\right]}_{i,i} & = & -\partial_{\mu_{\lambda_{i}}\mu_{\lambda_{i}}}E_{Q\left(\beta \right)}\left[\ln\;P\left(y,\beta ,\mu_{\lambda }\right)\right]\\ & = & {\left(\Pi_{\lambda }\right)}_{ii}-\frac{1}{2}tr\left(P_{i}\Sigma_{y}-P_{i}\Sigma_{y}P_{i}\Sigma_{y}\right)+\frac{1}{2}\epsilon_{y}^{T}P_{i}\epsilon_{y}+\frac{1}{2}tr\left(\Sigma_{\beta }J_{g}^{T}P_{i}J_{g}\right) \end{array}$$

Where $\Sigma_{y}=\Pi_{y}^{-1}$. The gradient ascent proceeds by alternately applying the two updates in Eq. (40). However, as mentioned above, a gradient ascent is rarely sufficient for robust performance, motivating an algorithm that dynamically switches between gradient ascent and Gauss-Newton-like updates. This is set out in the next section.

### 5.3 Updates in continuous time

Parameter updates in optimization are generally treated as occurring in discrete steps, $\Delta \mu_{\beta }$ and $\Delta \mu_{\lambda }$, as described above. However, Friston et al. (2007) considered updates occurring in continuous time, which provides a principled way to transition between gradient ascent and Gauss-Newton-like updates. In continuous time, the gradient ascent on the parameters can then be written in terms of the time derivative ${\dot{\mu }}_{\beta }\left(t\right)$ at time t:

(42) $${\dot{\mu }}_{\beta }\left(t\right)=\nabla_{\mu_{\beta }}\left[I_{\beta }\left(\mu_{\beta }\left[t\right]\right)\right]$$

Where $I_{\beta }\left(\mu_{\beta }\right)=E_{Q(\lambda )}\left[\ln\;P(y,\mu_{\beta },\lambda )\right]$. Integrating Eq. (42) over time using *local linearization* (Ozaki, 1985), the update for a time interval t is:

$$\Delta \mu_{\beta }=\left(\exp\left[t{\ddot{\mu }}_{\beta }\right]-I\right){\ddot{\mu }}_{\beta }^{-1}{\dot{\mu }}_{\beta }$$

(43) $${\ddot{\mu }}_{\beta }=\nabla_{\mu_{\beta }}\left[{\dot{\mu }}_{\beta }\right]=\partial_{\mu_{\beta }\mu_{\beta }}I_{\beta }\left(\mu_{\beta }\right)$$

And a similar expression is applied to update the hyperparameters. When t is small and positive, the algorithm behaves like a gradient ascent, because the term $\left(\exp[t{\ddot{\mu }}_{\beta }]-I\right)$ in Eq. (43) regularizes the update (i.e., reduces its size). This follows because the second derivatives ${\ddot{\mu }}_{\beta }$ are negative. As t increases, the matrix exponential term $\exp[t{\ddot{\mu }}_{\beta }]$ reduces to zero, resulting in a standard Gauss-Newton update (by around t=2):

(44) $$\Delta \mu_{\beta }=-{\ddot{\mu }}_{\beta }^{-1}{\dot{\mu }}_{\beta }$$

The integration time t can therefore be varied dynamically to adjust the behaviour of the algorithm. When the algorithm begins, the time is set to a small value, causing it to behave like a gradient scheme for stability. If the free energy has increased, i.e., improved as a result of an update, then regularization is decreased (by increasing t). Conversely, if the free energy has decreased, i.e., worsened, then regularization is increased (by decreasing t).

In practice, the step size *t* is generally specified with a (log) descent parameter v that is automatically scaled by the average curvature of the landscape α:

$$t=\frac{\exp\left(v\right)}{\alpha }$$

(45) $$\alpha =\exp\left[\frac{Re\left(\text{ln}\left|{\ddot{\mu }}_{\beta }\right|\right)}{n}\right]$$

Here, n is the number of parameters. A cautious, slow descent corresponds to v=−4 (the default starting value). As the average real eigenvalue of ${\ddot{\mu }}_{\beta }$ increases (i.e., the curvature increases), the regularisation is increased by decreasing v. The algorithm stops when the change in free energy becomes sufficiently small. In more detail, the predicted value of $I_{\beta }\left(\mu_{\beta }\right)$ after making the step $\Delta \mu_{\beta }$ is given by: $\nabla_{\mu_{\beta }}\left[I_{\beta }\left(\mu_{\beta }\right)\right]\cdot \Delta \mu_{\beta }$ . If this is small over a series of iterations, then the algorithm is considered to have converged. Pseudocode for the complete VL algorithm is provided in Appendix 2, and MATLAB code accompanies this article.

### 5.4 Interim summary

This section described an algorithm that searches for a probability density over the parameters Q(θ) that maximizes the free energy. The algorithm ascends the free energy landscape, with an adaptive step size. Readers experienced with machine learning may note some similarity with the Levenberg-Marquardt algorithm, however the continuous time approach reviewed here is derived from first principles without requiring the introduction of an arbitrary regularization term: see Friston et al. (2007) for a detailed comparison.

## 6 Examples

This section presents worked examples using simulated data, where the same algorithm introduced above is applied to different kinds of model. MATLAB code for each example is provided with this paper.

### 6.1 Static models

We start with a simple linear regression model with parameters β:

(46) y=Xβ+ϵ

For this example, the design matrix X has two columns: the first is a column of ones and the second consists of 100 evenly spaced values between −50 and 50. Thus, the parameter $\beta_{1}$ encodes the mean and $\beta_{2}$ encodes the regression slope. The observation noise is encoded by a single precision component, controlled by log-precision parameter λ:

$$\epsilon ∼N\left(0,\Pi_{y}^{-1}\right)$$

(47) $$\Pi_{y}=\exp\left(\lambda \right)I_{n}$$

**Fig. 5:**
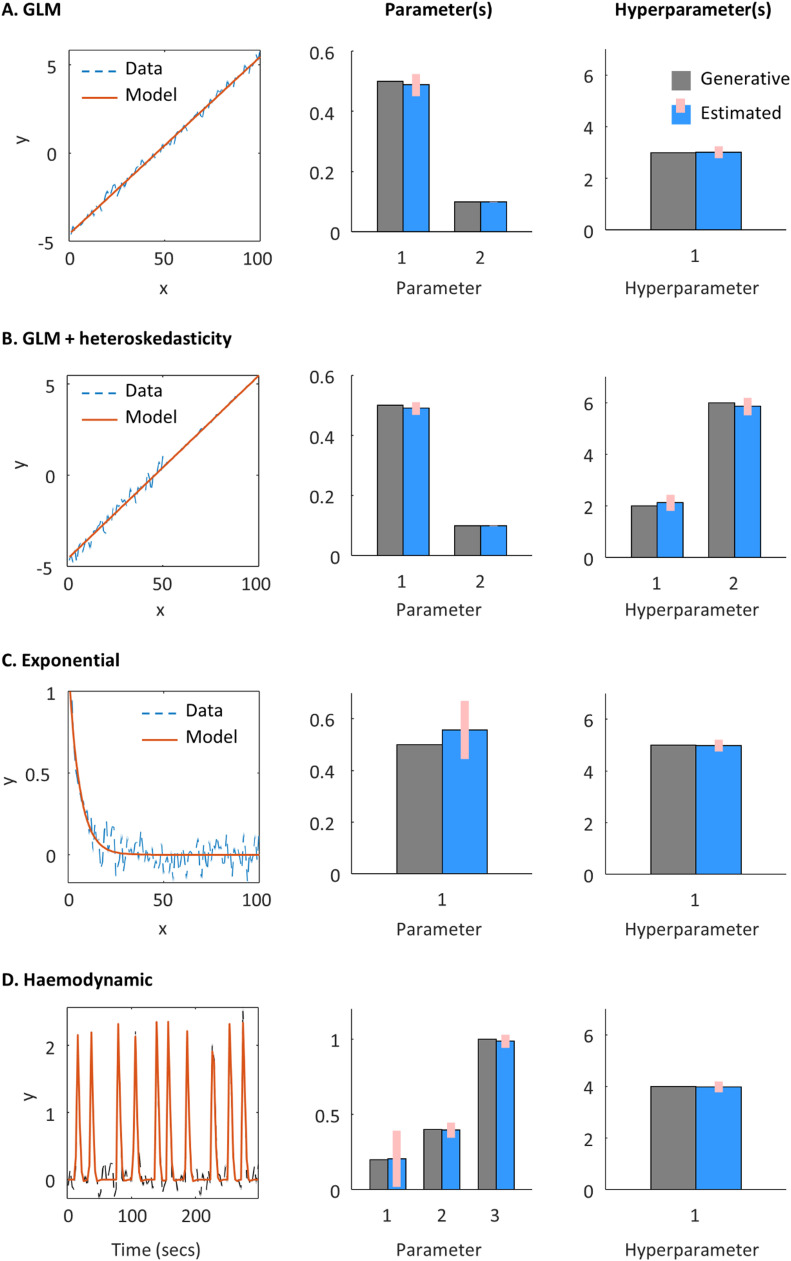
**Example applications of the VL scheme**. A-D illustrate the VL scheme applied to different models described in the text. **Left panels**: The simulated data (dashed blue lines) and prediction from the model (solid red lines). **Middle and right panels:** Parameter or hyperparameter values used to generate the data (grey bars without error bars) and posterior estimated values (blue bars with error bars).

Note that a truly linear model would converge within a single iteration of the VL scheme, whereas here there is an exponential function in Eq. (47), in order to ensure positivity of the hyperparameter λ. This introduces non-linearity into the model, thereby requiring several iterations to converge. Fig. 5A shows that the parameters and hyperparameters used to generate the simulated data (grey bars) were successfully recovered (blue bars with 90% credible intervals). Note that the covariance amongst (hyper)parameters was also calculated by the VL scheme, but is not shown.

The second example considers the situation where there is *heteroskedasticity* – observations having different levels of observation noise. This is a common situation, for example when parts of the data come from different measurement channels. This can be modelled by having two precision components:

(48) $$\Pi_{y}=\exp\left(\lambda_{1}\right)Q_{1}+\exp\left(\lambda_{2}\right)Q_{2}$$

Here, the first precision component matrix, $Q_{1}$ has values of one on the leading diagonal for the first half of the observations, and zeros for the second half. It therefore captures the precision of the first half of the observations. Similarly, $Q_{2}$ captures the precision of the second half of the observations. As shown in Fig. 5B, the parameters and hyperparameters used to generate the data were correctly recovered. Next, we illustrate dynamic models, i.e., those modelling continuous changes over time.

### 6.2 Dynamic models

A typical application of the VL algorithm is to estimate the parameters of dynamic models that are specified as ordinary differential equations (ODEs). As introduced in the empirical example in Section 1.1, this is accomplished by splitting the model into two parts: a model f of the dynamics of the unobserved (latent) variables x, and a static model g that translates the latent variables into observations y:

$$\dot{x}\left(t\right)=f\left(x\left(t\right),\beta \right)$$

(49) y(t)=g(x(t))+ϵ

Where x(t) is approximated using a standard numerical integration scheme, i.e.

(50) $$x\left(t\right)=\int_{0}^{t}\dot{x}\left(t\right)\;dt$$

Here, we perform this integration using the local linearization approach of Ozaki (1985). A simple example is estimating the rate of an exponential decay of a single observed variable x, with rate parameter β, for which the model specification corresponds to:

f(x(t),β)=−exp(β)x(t)

(51) g(x(t))=x(t)

The model fit and estimated parameters are shown in Fig. 5C.

Finally, we consider a more involved example – the haemodynamic model of Stephan et al. (2007), which is used in the analysis of functional magnetic resonance imaging (fMRI) data to infer neural dynamics. (This forms function g in Eq. (1), Section 1.1). For a detailed walkthrough of the physiology, please see Appendix 5 of Zeidman et al. (2019a). In brief, there are four hidden states (vasoactive signal s, blood inflow $f_{in}$, blood volume v, deoxyhaemoglobin q) and three time-invariant parameters that are estimated from the timeseries data (haemodynamic transit time $\tau_{h}$, vasoactive signal decay rate κ and stimulus efficacy z). The dynamics of the four hidden states are governed by the following equations, which together constitute f in Eq. (49):

(52) $$\begin{array}{ccc} {\dot{f}}_{in} & = & s\\ \dot{s} & = & z\left(t\right)-\kappa s-\gamma \left(f_{in}-1\right)\\ \tau_{h}\dot{v} & = & f_{in}\left(t\right)-f_{out}\left(v,t\right)\\ \tau_{h}\dot{q} & = & f_{in}\left(t\right)\frac{1-{\left(1-E_{0}\right)}^{\frac{1}{f_{in}\left(t\right)}}}{E_{0}}-\frac{f_{out}\left(v,t\right)q\left(t\right)}{v\left(t\right)} \end{array}$$

Where $f_{out}\left(v,t\right)=v{\left(t\right)}^{\frac{1}{\alpha }}$ and α,γ are fixed parameters. The final part of the model translates from the latent variables for blood flow v and deoxyhaemoglobin q to the fMRI timeseries y:

(53) $$\begin{array}{ccc} y & = & V_{0}\left(k_{1}\left(1-q\right)+k_{2}\left(1-\frac{q}{v}\right)+k_{3}\left(1-v\right)\right)\\ k_{1} & = & 4.3\cdot \vartheta_{0}\cdot E_{0}\cdot TE\\ k_{2} & = & \epsilon_{h}\cdot r_{0}\cdot E_{0}\cdot TE\\ k_{3} & = & 1-\epsilon_{h} \end{array}$$

Where for this example, $\vartheta_{0},E_{0},r_{0},TE,\epsilon_{h}$ are fixed parameters. Fig. 5D shows that the parameters were recovered successfully, although the precision with which the transit time parameter could be recovered was lower than the other two parameters, as reflected in the larger 90% credible interval (pink bar).

### 6.3 Bayesian model comparison

The main purpose of the VL scheme is to enable Bayesian model comparison, which is comparing the relative evidence for different models, where the log evidence is approximated by the free energy. If each model encodes a hypothesis for how the data were generated, then Bayesian model comparison enables different hypotheses to be compared, in terms of how well they trade off accuracy and complexity (see Section 3.2).

Models to be compared can differ in their likelihood – i.e., the definition of their forward model − and/or in the specification of their priors. The only requirement is that all models have been fitted to the same data (where this is not the case, an alternative approach referred to as *Bayesian Data Comparison* may be considered, see Zeidman et al. (2019c)). Where models differ only in their priors, there is no need to fit each model separately to the data using the VL scheme – it is sufficient to fit one ‘full’ model, and use *Bayesian model reduction* to analytically compute the free energy and parameters of the alternative reduced models, under Laplace approximations (Friston et al., 2018). Through these methods, decisions such as whether to include particular variables in the model or how to capture their interactions can be decided in a principled manner.

To illustrate this, we will use GLM example above, where the first half of the observations were noisier than the second half (illustrated in Fig. 5B, dashed lines, left panel). We will then use Bayesian model comparison to ask which is the best explanation for the data: a model with one precision component (i.e., all observations had the same level of noise), two precision components (the first and second half of the observations had different levels of observation noise) or three components (each third of the data had a different level of noise). Including more precision components could increase the model's accuracy by introducing more degrees of freedom, but would also increase model complexity. To assess which of the three models optimized the accuracy / complexity trade-off, we specified each model and calculated their free energy using the VL scheme. The values were $F_{1}=-18.21,\;F_{2}=39.61,\;F_{3}=14.32$, where a more positive free energy is better.

These free energies can then be compared in terms of the *Bayes factor* (Kass and Raftery, 1995) and posterior probabilities. The Bayes factor is the ratio of evidence for a model $m_{1}$ relative to another model $m_{2}$:

(54) $$B_{1}=\frac{p\left(y|m_{1}\right)}{p\left(y|m_{2}\right)}$$

The larger the ratio, stronger the evidence in favour of model $m_{1}$. Taking the log, division becomes subtraction, therefore the log Bayes factor is simply the difference in log evidence. The log Bayes factor in favour of model 1 is:

(55) $$\ln\;B_{1}=\ln\;p\left(y|m_{1}\right)-\ln\;p\left(y|m_{2}\right)\approx F_{1}-F_{2}$$

**Fig. 6:**
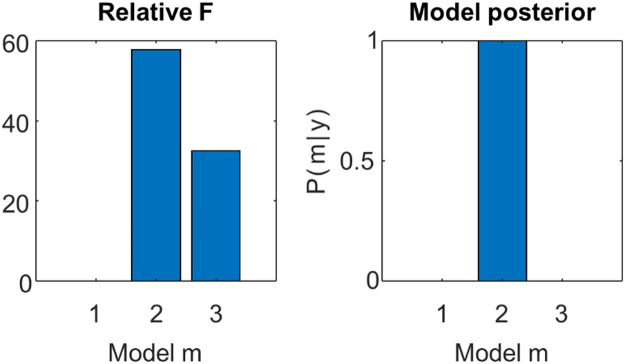
**Bayesian model comparison**. Three general linear models (GLMs) were fitted to the data using the VL scheme, and then compared based on their free energy. The models differed in whether they had one, two or three precision components. **Left:** The log Bayes factor for each model relative to model 1. **Right:** The same results transformed to a posterior probability for each model.

The log Bayes factor can be computed for more than two models by selecting a model to serve as the baseline or reference. Here, we chose the worst model, $m_{1}$, as the reference model, and calculated log Bayes factor for models $m_{2}$ and $m_{3}\;$ relative to $m_{1}$, as shown in Fig. 6 (left panel). As expected, $m_{2}$ was the best (as the data were generated using two precision components), $m_{3}$ was the second best, and $m_{1}$ was the worst model. This example also makes a key point, that accuracy is not an apt measure of model quality. The third model here would have been the most accurate because it has more degrees of freedom. However, its added complexity was correctly penalized by the free energy, ensuring it would be discarded in favour of the model that is the simplest explanation for the data – but not too simple.

It can aid interpretation to report not only the log Bayes factor, but also the posterior probability for each model, e.g., $p(m_{1}|y)$. Under equal prior probability for each model, by application of Bayes rule, the posterior probabilities are given by a softmax function of the log Bayes factors:

(56) $$\begin{array}{ccc} p\left(m_{i}|y\right) & = & \frac{p\left(y|m_{i}\right)}{p\left(y\right)}\\ & = & \frac{1}{1+\exp\left(-\ln\;B_{i}\right)} \end{array}$$

This is illustrated in Fig. 6 (right panel). This demonstrates that $m_{2}$ had posterior probability close to unity, meaning that we could be extremely confident that it provided the best explanation for the data. This procedure may be applied with any number of models, and therefore forms the basis for hypothesis testing in Bayesian inference.

## 7 Empirical example of Bayesian model comparison

In Section 2 we introduced an example modelling problem, where the aim was to compare the evidence for two candidate models, as explanations for why visual region V5 of the brain is enhanced by visual attention. Here, we illustrate applying the VL scheme to the two candidate models, $m_{1}$ and $m_{2}$ and performing Bayesian model comparison.

**Fig. 7:**
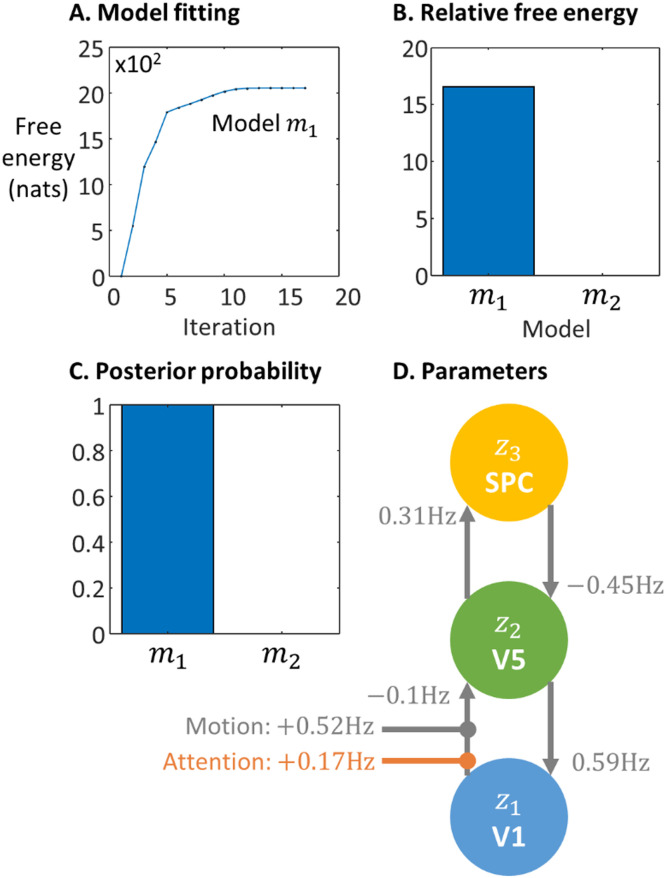
**Results of applying VL to the fMRI attention example. A.** The free energy per iteration of the VL model estimation scheme for model $m_{1}$, relative to the free energy of the first iteration (i.e., this shows the increasing log Bayes factor). **B.** The free energy per model relative to $m_{2}$ which was set to zero. **C.** The posterior probability for each model. **D.** The expected values of the parameters. With reference to Appendix 1, which details the parameterisation of the model - numbers on the connections relate to parameter matrix A of the model, whereas the effects of motion and attention relate to the parameter matrices B of the model.

Fig. 7A shows the free energy over iterations for model $m_{1}$ (relative to the first iteration). The algorithm converged after 17 iterations. The resulting free energies for the two models were $F_{1}=-3277.61$ and $F_{2}=-3294.20$ respectively, where a more positive free energy is better. These free energies were then taken forward for Bayesian model comparison.

The log Bayes factor was $\ln B_{1}=F_{1}-F_{2}=15.59$. Taking the exponential to undo the log, this means there was exp(15.59)=5,897,269 times the evidence for $m_{1}$ than $m_{2}$ (Fig. 7B). Naturally, therefore, the posterior probability in favour of $m_{1}$ approached unity (Fig. 7C).

Having reported the probability for each model, studies typically report the posterior estimates of the parameters from the winning model if there's a clear winner, or alternatively if there's no clear winner, the (precision-weighted) average of parameters across models (this is called Bayesian model averaging). Fig. 7D shows the posterior expected values from model $m_{1}$. It can be seen that under this model, the presence of visual motion boosted the connection from brain region V1 to V5 (by 0.52 Hz), and attention to visual motion further increased the strength of this connection (by 0.17 Hz).

Together, one may conclude that the data were best explained by hypothesis H1 – i.e., attentional modulation of V5 could be accounted for by bottom-up connectivity from V1. Examining the estimated parameters of the model demonstrated that attention had a gating effect on feed-forward or ascending connections from primary visual cortex.

## 8 Discussion

The VL scheme described here underwrites thousands of neuroimaging studies, and is beginning to find applications in other fields. We considered it important, therefore, to clearly explain how it works, what assumptions it makes, and how to interpret the outputs. To this end, Sections 1-3 set out the key challenge of Bayesian inference – the intractable integral within the model evidence (Eq. (3)) – and how this can be resolved using variational Bayes (a.k.a., approximate Bayesian inference). This involves defining a lower bound on the log evidence (the free energy, Eq. (11)), and then identifying a probability density over the parameters that maximizes this bound, bringing it as close as possible to the log evidence. In effect, variational procedures convert an impossible integration or marginalisation problem into a tractable optimisation problem. Section 4 set out the implementation of variational Bayes typically used in neuroimaging (Eq. (31)), where Laplace approximations (i.e., normal densities) are used to approximate the free energy bound. Section 5 then described an efficient algorithm for maximizing this (approximate) free energy, as illustrated for static and dynamic models in Section 6. Worked derivations are provided in the supplementary materials, pseudocode is provided in the appendix and standalone MATLAB code accompanies this article.

This scheme has several advantages over alternative methods, both in terms of the free energy approximation of the log evidence and the algorithm used to maximize it. First, the free energy serves as a better approximation of the log evidence than other commonly used heuristics like the AIC and BIC. These approximations can fail dramatically even in relatively benign settings – see (Penny, 2012a) for some unsettling examples. While all three measures can be decomposed into accuracy and complexity terms, only the free energy takes into account uncertainty in the parameters for the accuracy term, as well as the covariance amongst parameters in the complexity term (Penny, 2012b). VL also has advantages over non-variational sampling methods. While sampling is highly effective for profiling the shape of a probability density, it does not provide a straightforward way to estimate the log evidence (one common approach, the *harmonic mean*, has been described as the “Worst Monte Carlo Method Ever” for its poor performance^9^). The lower computational cost of VL relative to sampling schemes, together with the fact that it is deterministic (so always provides the same results given the same data) provide further advantages. Regarding the algorithm described here for maximizing the free energy, the closest alternative is Expectation Maximization (EM). EM differs from variational Bayes in that EM ignores uncertainty about the hyperparameters. Thus, an advantage of VL is that it conveys the uncertainty of both the parameters and hyperparameters to the next iteration of the algorithm (i.e., EM is a special case of the variational Bayes where uncertainty about the hyperparameters is ignored.)

There are potential drawbacks of the VL scheme. First, the Laplace assumption may not be suitable for all applications. For instance, a Gaussian posterior may not be appropriate where the true posterior is multimodal, if a multimodal posterior is important for making inferences. To evaluate this for a particular application, the validity of the Laplace assumption can be assessed using sampling methods. This can be particularly useful when dealing with highly nonlinear models. Typically, variational Laplace accommodates nonlinearities by applying gaussian assumptions to nonlinear transformations of the parameters. A nice example of this is the treatment of hyperparameters above Eqs. (47)-(48). By taking the exponential of the hyperparameter, one is effectively assuming a log normal prior, which ensures positivity for scale parameters of this sort (a scale parameter is a nonnegative parameter, such as a rate or time constant, variance or distance measure). Using the same device in hierarchical and nonlinear models allows one to accommodate a large range of (weakly) non-linear models within variational Laplace. However, it is sometimes necessary to check the robustness to violations of the Laplace assumption with reference to sampling schemes.

Second, the algorithm presented here is highly likely to converge to an optimal value, but only if the initial values of the parameters are well chosen (typically, the prior expected values of the parameters are used). In other words, if there are multiple local optima, then the algorithm may not be able to escape the local optimum that is easiest to reach from the starting value (this is sometimes expressed as starting within the basin of attraction of a fixed point in the free energy landscape). To overcome this, multi-start approaches have been used in conjunction with the VL scheme. For example, in the analysis of neuroimaging data from multiple test subjects, a common approach is to iteratively restart the algorithm from the group average parameter values (Friston et al., 2015). Finally, it should be noted that variational Bayes methods commonly suffer from overconfidence in their posterior parameter estimates, as demonstrated in the context of DCM for fMRI by Daunizeau et al. (2012).

Various extensions and variants of the VL scheme have been developed to handle a broader range of models in the context of neuroimaging, which we have not had space to detail in this article. For example, VL has been applied to modelling data in the frequency domain (complex cross-spectra), which is routinely used in the analysis of electrophysiological data (Moran et al., 2009) and resting state fMRI data (Friston et al., 2014). Similar approaches have been introduced to invert stochastic differential equation models, namely Dynamic Expectation Maximization (DEM) and Generalised Filtering (GF), which estimate a model's hidden states and parameters, treating both as time-dependant random variables (Friston et al., 2010; Friston et al., 2008b). VL has also been applied to model voxel-wise fMRI data, yielding maps of parameters and posterior probabilities (Zeidman et al., 2018; Puckett et al., 2020). That approach leveraged parallelisation to estimate multiple voxels’ timeseries independently; future work could improve performance by sharing parameters across voxels, such as those relating to haemodynamics or observation noise. More recently, the Parametric Empirical Bayes (PEB) framework was introduced to extend the VL scheme to hierarchical experimental designs, where for example, data have been sampled from multiple subjects at multiple time points (Zeidman et al., 2019b; Friston et al., 2016).

Since the introduction of the VL scheme, other algorithms and software tools for variational Bayesian inference have been introduced that serve a similar role. The Variational Message Passing (VMP) algorithm pre-dates VL (Winn et al., 2005) and is now used in the Microsoft infer.NET programming language and the ForneyLab Julia package (Cox et al., 2019). Like VL, this is a deterministic algorithm, which inverts models that can be expressed as *Bayesian networks* or *Forney Factor Graphs* (with the prerequisite that nodes are conjugate to their parent). The distributed nature of the VMP algorithm has also enabled its use as a model for how inference is performed in biological neural networks (Parr et al., 2019). Another prominent algorithm is automatic differentiation variational inference (ADVI) (Kucukelbir et al., 2017), which is implemented in multiple probabilistic programming frameworks including Stan (Kucukelbir et al., 2015), Turing.jl for Julia (Ge et al., 2018), PyMC3 for Python and Tensorflow Probability (TFP). Like VL, ADVI optimizes the free energy, however this is performed without Laplace approximations of the free energy functional. Instead, the log joint in Eq. (11) is evaluated automatically from a given graphical model, and the expected value is approximated using Monte Carlo integration methods, when the free energy is evaluated. Having defined the free energy functional, the next step is to maximize it, and VL and ADVI differ in how they do this. In VL, the gradient of the free energy under the Laplace approximation is given by Eq. (40), which depends on first computing the gradient of the function to optimized, g(θ), using numerical methods (finite differences). ADVI estimates the gradient of the free energy using Automatic differentiation (AD), which involves reducing the free energy into a graph of elemental math operations and then repeatedly applying the chain rule (Griewank, 1989; Iri, 1984). These gradients are then supplied to a stochastic gradient descent algorithm (Hoffman et al., 2013). An interesting future direction would be to compare the performance of the VL scheme against VMP and ADVI for the kind of models typically applied in neuroimaging. We would predict that VL and VMP would be significantly faster because they eschew sampling, however ADVI would offer the most accurate posteriors in situations where the Laplace approximation is violated. Finally, VL and ADVI may also be compared against a recently developed scheme called Stochastic VB (sVB), which was introduced in the neuroimaging literature and also features stochastic gradient descent. It has been applied to discovering functional modes in neuroimaging data using hidden Markov models (Vidaurre et al., 2017) and quantifying variability in resting state networks across a large sample of the population in the UK Biobank (Farahibozorg et al., 2021).

The code accompanying this paper illustrates applications of the VL scheme with a variety of models. Readers interested in learning more about the applications of VL may wish to proceed to recent tutorials on modelling neuroimaging data using dynamic causal modelling (DCM) (Zeidman et al., 2019a) and behavioural data using active inference (Smith et al., 2022).

## 9 Code availability

MATLAB code accompanying this paper can be downloaded from https://github.com/pzeidman/vl-tutorial.

## 10 Data and code availability

All MATLAB code accompanying this paper can be downloaded from Github at https://github.com/pzeidman/vl-tutorial.

## Declaration of Competing Interest

The authors declare that they have no known competing financial interests or personal relationships that could have appeared to influence the work reported in this paper.

## Figures and Tables

**Table:** 

Inputs:
Generative model$g(\mu_{\beta })$
Data vectory
Starting values for (hyper)parameters$\mu_{\beta }$and$\mu_{\lambda }$
Prior expected values$\eta_{\beta }$and$\eta_{\lambda }$
Prior precision matrices$\Pi_{\beta }$and$\Pi_{\lambda }$
Precision component matrices$Q_{1}\ldots Q_{k}$
Outputs:
Posterior expected values$\mu_{\beta }$and$\mu_{\lambda }$
Posterior covariances$\Sigma_{\beta }$and$\Sigma_{\lambda }$
Data precision matrix$\Pi_{y}$
Free energyF
// Initialize to a high level of regularization
*v*= −4
until convergence
// Compute numerical derivative of g wrt parameters
${\left(J_{g}\right)}_{ij}=\partial_{\mu_{j}}g_{i}\left(\mu_{\beta }\right)$
/* Optionally, if the Jacobian is unstable, make small parameter updates and re-try computing the Jacobian */
// Optimize hyperparameters
until convergence
// Compute data precision$\Pi_{y}$and data precision per component$P_{i}$
$P_{i}=\exp{\left(\mu_{\lambda }\right)}_{i}Q_{i}$
$\Pi_{y}=\sum_{i}P_{i}$
// Compute posterior covariance over parameters
$\Sigma_{\beta }={\left(J_{g}^{T}\Pi_{y}J_{g}+\Pi_{\beta }\right)}^{-1}$
// Compute error terms
$\epsilon_{\lambda }=\mu_{\lambda }-\eta_{\lambda }$
$\epsilon_{y}=y-g\left(\mu_{\beta }\right)$
// Compute 1st and 2nd derivatives of the expected hyperparameters wrt g
${\dot{\mu }}_{\lambda }=\partial_{\mu_{\lambda_{i}}}E_{Q(\beta )}\left[\ln P(y,\beta ,\lambda )\right]$
$=\frac{1}{2}tr\left(P_{i}\Pi_{y}^{-1}\right)-\frac{1}{2}\epsilon_{y}^{T}\;P_{i}\epsilon_{y}-\partial_{\mu_{\lambda_{i}}}{\left(\epsilon_{\lambda }\right)}^{T}\Pi_{\lambda }\epsilon_{\lambda }-\frac{1}{2}tr\left(\Sigma_{\beta }J_{g}^{T}\;P_{i}J_{g}\right)$
${\ddot{\mu }}_{\lambda }=\partial_{\mu_{\lambda_{i}}\mu_{\lambda_{i}}}E_{Q(\beta )}\left[\ln P(y,\beta ,\lambda )\right]$
$=-{\left(\Pi_{\lambda }\right)}_{ii}+\frac{1}{2}tr\left(P_{i}\Sigma_{y}-P_{i}\Sigma_{y}P_{i}\Sigma_{y}\right)-\frac{1}{2}\epsilon_{y}^{T}P_{i}\epsilon_{y}-\frac{1}{2}tr\left(\Sigma_{\beta }J_{g}^{T}P_{i}J_{g}\right)$
// Scale a relaxed regularization value (*v* = 4) by the curvature
$t\;=\exp(4\;-Re(\text{ln}|{\ddot{\mu }}_{\lambda }|)/n)$
// Update hyperparameters
$\mu_{\lambda }=\mu_{\lambda }+\left(\exp[t\times {\ddot{\mu }}_{\lambda }]-I\right){\ddot{\mu }}_{\lambda }^{-1}{\dot{\mu }}_{\lambda }\left(t\right)$
end
// Calculate free energy
*F*= … // (see Eq. (30))
// Assess performance
if F has improved or we're early in the optimization then
// Compute errors
$\epsilon_{\beta }=\mu_{\beta }-\eta_{\beta }$
$\epsilon_{y}=y-g\left(\mu_{\beta }\right)$
/* Compute 1st and 2nd derivatives of the expected parameters wrt g for parameter update */
${\dot{\mu }}_{\beta }=J_{g}^{T}\Pi_{y}\epsilon_{y}-\Pi_{\beta }\epsilon_{\beta }$
${\ddot{\mu }}_{\beta }=-J_{g}^{T}\Pi_{y}J_{g}-\Pi_{\beta }$
// Decrease regularization
*v* = *v*+ 1/2
else
// Free energy has got worse
$\mu_{\beta }$**,**$\mu_{\lambda }\leftarrow$restore previous parameter estimates
// Increase regularization
*v* = v - 2
end
// Scale the log-regularization v by the curvature
$t\;=\exp(v\;-Re(\text{ln}|{\ddot{\mu }}_{\beta }|)/n)$
// Update parameters
$\mu_{\beta }=\mu_{\beta }+\left(\exp[t\;\times {\ddot{\mu }}_{\beta }]-I\right){\ddot{\mu }}_{\beta }^{-1}{\dot{\mu }}_{\beta }$
if the change in F is repeatedly below criterion
// convergence
Return
end
End

## Data Availability

No data was used for the research described in the article.

## Appendix 1 DCM for fMRI

This appendix details the parameterisation of connectivity matrices used in the attention to visual motion example. The neural connectivity matrix in Eq. (1) is defined as:

$$J\left(t\right)≔\left(A+u_{2}\left(t\right)B^{\left(\mathrm{MOTION}\right)}+u_{3}\left(t\right)B^{\left(\mathrm{ATTENTION}\right)}\right)$$

Where binary indicator variables $u_{2}\left(t\right)$ and $u_{3}\left(t\right)$ denote, respectively, whether stimuli were in motion at time t and whether participants were instructed to pay attention to speed of the motion (Fig. 1A, top). The other terms are matrices of connectivity parameters, which have units of Hertz ($s^{-1}$) and need to be estimated from the data. For the first hypothesis, H1, the connectivity matrices are parameterised as follows:

$$A≔\left[\begin{array}{ccc} a_{11} & a_{12} & 0\\ a_{21} & a_{22} & a_{32}\\ 0 & a_{23} & a_{33} \end{array}\right]$$

$$B^{\left(\mathrm{MOTION}\right)}≔\left[\begin{array}{ccc} 0 & 0 & 0\\ b_{21}^{\left(MOTION\right)} & 0 & 0\\ 0 & 0 & 0 \end{array}\right]$$

$$B^{\left(\mathrm{ATTENTION}\right)}≔\left[\begin{array}{ccc} 0 & 0 & 0\\ b_{21}^{\left(ATTENTION\right)} & 0 & 0\\ 0 & 0 & 0 \end{array}\right]$$

In detail, matrix A encodes the coupling strength amongst brain regions, where element $a_{i,j}$ is the strength of the connection from region j to region i in units of hertz ($s^{-1})$. Thus, the columns of matrix A correspond to outgoing connections from regions V1, V5 and SPC, and the rows correspond to incoming connections. Parameters $b_{21}^{\left(MOTION\right)}$ and $b_{21}^{\left(ATTENTION\right)}$ encode the change in the V1→V5 connection due to motion and attention respectively. Finally, parameter matrix C encodes the sensitivity of each brain region to the driving effect of visual stimuli to region V1:

$$C≔\left[\begin{array}{ccc} c_{11} & 0 & 0\\ 0 & 0 & 0\\ 0 & 0 & 0 \end{array}\right]$$

A model corresponding to the second hypothesis, H2, may be specified through a minor modification to the parameter matrix $B^{\left(\mathrm{ATTENTION}\right)}$, to indicate that attention should modulate the SPC→V5 connection rather than the V1→SPC connection:

$$B^{\left(\mathrm{ATTENTION}\right)}≔\left[\begin{array}{ccc} 0 & 0 & 0\\ 0 & 0 & b_{23}^{\left(ATTENTION\right)}\\ 0 & 0 & 0 \end{array}\right]$$

This second model is illustrated in Fig. 1B (right). In practice, the elements of these parameter matrices were either switched on (i.e., informed by the data) or switched off (fixed at zero) by selecting appropriate prior means and variances.

## Appendix C Supplementary materials

Image, application 1

## Footnotes

1 These models are used as teaching examples, which we supply with the SPM software package. The dataset and analysis scripts, can be freely downloaded from https://www.fil.ion.ucl.ac.uk/spm/data/attention/ .

2 A *functional* is a function of a function. More specifically - at least for the purposes of this paper - it is a mapping whose input is a function and whose output is a number.

3 This routine is particularly useful for fitting continuous data when the likelihood has a Gaussian form (whose expectation and covariance may be non-linear functions of the parameters). An alternative MATLAB function, spm_nlsi_Newton, allows the specification of generic likelihood functions (for example, it could be used for fitting binary data using likelihood densities compatible with logistic regression models).

4 The following mathematical notation is used. **Variables**: Lower case italic text (x) is used for scalar variables, bold uppercase letters (Y) are used for matrices and bold lower-case letters are used for vectors (y). The expected value or average of variable x under the density Q is written $E_{Q}\left[x\right]$. **Calculus**: The partial derivative of a function h(x) with respect to x is written $\partial_{x}\;h\left(x\right)$, therefore $\partial_{x}=\frac{\partial }{\partial_{x}}$ . The second derivative of h(x) is written $\partial_{xx}\;h\left(x\right)$. **Variational calculus:** The *variation* of a functional δ may be thought of as the derivative of the functional with respect to the function. For instance, for a functional of the form A=∫S(h(x))dx, the variation of A with respect to h is: $\delta_{h(x)}S$.

5 For a probability density function P(x), the expected value E[P(x)] is a weighted average over the possible values of x, where each value is weighted by its probability, i.e., E[P(x)]=∫xP(x)dx. The expected value can also be taken with respect to another probability density function Q(x), which we write as $E_{Q(x)}\left[P(x)\right]=\int Q\left(x\right)P\left(x\right)dx$. This is a weighted average of P(x), where the weighting comes from Q(x).

6 Note that this step ensures the final result is the same regardless of whether μ is the mode of P(θ|y) (prior to optimisation of the approximate posterior, it may not be). The linear term in the expansion will still disappear under the expectation as it includes a factor of $E_{Q(\theta )}\left[\theta -\mu \right]$ = 0, recovering Eq. 21.

7 In the canonical implementation of the VL scheme (spm_nlsi_gn), the Jacobian is the negative of that defined here. For this reason, the sign of various terms is switched here compared to the original implementation.

8 An important point here is that Σ is treated as constant with respect to the posterior expectation (seemingly contradicting the idea that the former is an analytic function of the latter). This is consistent with the Laplace approximation, as the assumption that the log joint is approximately quadratic implies that its curvature (and therefore the posterior covariance) is constant.

9 https://radfordneal.wordpress.com/2008/08/17/the-harmonic-mean-of-the-likelihood-worst-monte-carlo-method-ever/
