# Supplementary material for: A primer on Variational Laplace (VL)
Source: Neuroimage. 2023 Oct 1;279:120310. doi: 10.1016/j.neuroimage.2023.120310 (PMC10951963; doi:10.1016/j.neuroimage.2023.120310)
Supplement: Supplementary file 1 [file mmc1.pdf]

Supplementary: Derivation of variational updates

Zeidman, Friston, Parr, *A primer on Variational Laplace (VL)* (2023)

These supplementary materials provide worked derivations of many of the equations in the main text. We begin with some fundamental identities which are used repeatedly through the paper – the gradients and second derivatives of the log joint distribution $\ln P(\boldsymbol{y}, \boldsymbol{\beta}, \boldsymbol{\lambda})$.

1. Key gradients and derivatives

1.1 Gradient of the log joint w.r.t. parameters

From Eq 31, the log joint is:

$$\ln P(\boldsymbol{y}, \boldsymbol{\beta}, \boldsymbol{\lambda}) = \underbrace{-\frac{1}{2}\left[\ln\left|\boldsymbol{\Pi}_{\mathbf{y}}^{-1}\right| + \boldsymbol{\epsilon}_{\boldsymbol{y}}^{T}\boldsymbol{\Pi}_{\mathbf{y}}\boldsymbol{\epsilon}_{\boldsymbol{y}}\right]}_{\text{Likelihood}} \underbrace{-\frac{1}{2}\left[\ln\left|\boldsymbol{\Pi}_{\boldsymbol{\beta}}^{-1}\right| + \boldsymbol{\epsilon}_{\boldsymbol{\beta}}^{T}\boldsymbol{\Pi}_{\boldsymbol{\beta}}\boldsymbol{\epsilon}_{\boldsymbol{\beta}}\right]}_{\text{Prior (parameters)}} \underbrace{-\frac{1}{2}\left[\ln\left|\boldsymbol{\Pi}_{\boldsymbol{\lambda}}^{-1}\right| + \boldsymbol{\epsilon}_{\boldsymbol{\lambda}}^{T}\boldsymbol{\Pi}_{\boldsymbol{\lambda}}\boldsymbol{\epsilon}_{\boldsymbol{\lambda}}\right]}_{\text{Prior (hyperparameters)}} \quad \textit{(S1)}$$

Dropping terms that do not depend on the parameters $\boldsymbol{\beta}$, and evaluating at the (approximate) posterior modes:

$$\nabla_{\mu_\beta} \ln P(\boldsymbol{y}, \boldsymbol{\mu_\beta}, \boldsymbol{\mu_\lambda}) = \nabla_{\mu_\beta}\left[-\frac{1}{2}\boldsymbol{\epsilon}_{\boldsymbol{\beta}}^{T}\boldsymbol{\Pi}_{\boldsymbol{\beta}}\boldsymbol{\epsilon}_{\boldsymbol{\beta}}\right] + \nabla_{\mu_\beta}\left[-\frac{1}{2}\boldsymbol{\epsilon}_{\boldsymbol{y}}^{T}\boldsymbol{\Pi}_{\mathbf{y}}\boldsymbol{\epsilon}_{\boldsymbol{y}}\right] \quad \textit{(S2)}$$

Tackling the first term in element-wise fashion:

$$\begin{aligned}
\left(\nabla_{\boldsymbol{\mu_\beta}}\left[-\frac{1}{2}\boldsymbol{\epsilon}_{\boldsymbol{\beta}}^{T}\boldsymbol{\Pi}_{\boldsymbol{\beta}}\boldsymbol{\epsilon}_{\boldsymbol{\beta}}\right]\right)_i &= -\frac{1}{2}\partial_{\mu_{\beta_i}}\sum_j\sum_k \epsilon_{\beta_j}\epsilon_{\beta_k}\Pi_{\beta_{jk}} \\
&= -\frac{1}{2}\sum_j\sum_k \partial_{\mu_{\beta_i}}\left(\epsilon_{\beta_j}\epsilon_{\beta_k}\Pi_{\beta_{jk}}\right) \\
&= -\frac{1}{2}\sum_j\sum_k \partial_{\mu_{\beta_i}}\left(\epsilon_{\beta_j}\right)\epsilon_{\beta_k}\Pi_{\beta_{jk}} - \frac{1}{2}\sum_j\sum_k \partial_{\mu_{\beta_i}}\left(\epsilon_{\beta_k}\right)\epsilon_{\beta_j}\Pi_{\beta_{jk}} \\
&= -\partial_{\mu_{\beta_i}}\left(\boldsymbol{\epsilon_\beta}\right)^{\mathrm{T}}\boldsymbol{\Pi_\beta}\boldsymbol{\epsilon_\beta}
\end{aligned} \quad \textit{(S3)}$$

$$\Rightarrow \nabla_{\boldsymbol{\mu_\beta}}\left[-\frac{1}{2}\boldsymbol{\epsilon}_{\boldsymbol{\beta}}^{T}\boldsymbol{\Pi}_{\boldsymbol{\beta}}\boldsymbol{\epsilon}_{\boldsymbol{\beta}}\right] = -\partial_{\mu_\beta}\left(\boldsymbol{\epsilon_\beta}\right)^{\mathrm{T}}\boldsymbol{\Pi_\beta}\boldsymbol{\epsilon_\beta} = -\boldsymbol{\Pi_\beta}\boldsymbol{\epsilon_\beta}$$

Similarly, for the second term:

$$\nabla_{\mu_\beta}\left[-\frac{1}{2}\boldsymbol{\epsilon}_{\boldsymbol{y}}^{T}\boldsymbol{\Pi}_{\boldsymbol{y}}\boldsymbol{\epsilon}_{\boldsymbol{y}}\right] = -\partial_{\mu_\beta}\left(\boldsymbol{\epsilon_y}\right)^{\mathrm{T}}\boldsymbol{\Pi_y}\boldsymbol{\epsilon_y} = g'(\boldsymbol{\mu_\beta})\boldsymbol{\Pi_y}\boldsymbol{\epsilon_y} = \mathbf{J}_{\boldsymbol{g}}^{T}\boldsymbol{\Pi_y}\boldsymbol{\epsilon_y} \quad \textit{(S4)}$$

Where $\boldsymbol{J_g}$ is the Jacobian matrix of first partial derivatives, with $(\mathbf{J}_{\boldsymbol{g}})_{ij} = \partial_{\mu_j} g_i(\boldsymbol{\mu_\beta})$ for observation i and parameter j.

Putting the two terms together:

$$\nabla_{\mu_\beta} \ln P(\boldsymbol{y}, \boldsymbol{\mu_\beta}, \boldsymbol{\mu_\lambda}) = \mathbf{J}_{\boldsymbol{g}}^{T}\boldsymbol{\Pi_y}\boldsymbol{\epsilon_y} - \boldsymbol{\Pi_\beta}\boldsymbol{\epsilon_\beta} \quad \textit{(S5)}$$

1.2 Gradient of the log joint w.r.t. hyperparameters

The data precision matrix is a linear mixture of precision components $\boldsymbol{Q_1} \ldots \boldsymbol{Q_k}$, each weighted by the exponential of a hyperparameter $\boldsymbol{\lambda} = (\lambda_1 \ldots \lambda_k)$. Re-writing the log joint to include only terms that depend on $\boldsymbol{\lambda}$, the partial derivative with respect to $\boldsymbol{\lambda}$ is:

$$\nabla_{\mu_\lambda} \ln P(\boldsymbol{y}, \boldsymbol{\mu_\beta}, \boldsymbol{\mu_\lambda}) = \nabla_{\mu_\lambda}\left[-\frac{1}{2}\boldsymbol{\epsilon}_{\boldsymbol{\lambda}}^T \boldsymbol{\Pi}_{\boldsymbol{\lambda}} \boldsymbol{\epsilon}_{\boldsymbol{\lambda}}\right] + \nabla_{\mu_\lambda}\left[-\frac{1}{2}\ln\left|\boldsymbol{\Pi}_{\mathbf{y}}^{-\mathbf{1}}\right|\right] + \nabla_{\mu_\lambda}\left[-\frac{1}{2}\boldsymbol{\epsilon}_{\boldsymbol{y}}^T \boldsymbol{\Pi}_{\mathbf{y}} \boldsymbol{\epsilon}_{\boldsymbol{y}}\right] \tag{S6}$$

The first term is:

$$\begin{aligned}\left(\nabla_{\mu_\lambda}\left[-\frac{1}{2}\boldsymbol{\epsilon}_{\boldsymbol{\lambda}}^T \boldsymbol{\Pi}_{\boldsymbol{\lambda}} \boldsymbol{\epsilon}_{\boldsymbol{\lambda}}\right]\right)_i &= -\frac{1}{2}\sum_j \sum_k \partial_{\mu_{\lambda_i}}\left(\epsilon_{\lambda_j}\epsilon_{\lambda_k}\Pi_{\lambda_{jk}}\right) \\ &= -\frac{1}{2}\sum_j \sum_k \partial_{\mu_{\lambda_i}}\left[\epsilon_{\lambda_j}\right]\epsilon_{\lambda_k}\Pi_{\lambda_{jk}} + \epsilon_{\lambda_j}\partial_{\mu_{\lambda_i}}\left[\epsilon_{\lambda_k}\right]\Pi_{\lambda_{jk}} + \epsilon_{\lambda_j}\epsilon_{\lambda_k}\partial_{\mu_{\lambda_i}}\left[\Pi_{\lambda_{jk}}\right] \\ &= -\partial_{\mu_{\lambda_i}}(\boldsymbol{\epsilon}_{\boldsymbol{\lambda}})^{\mathrm{T}} \boldsymbol{\Pi}_{\boldsymbol{\lambda}} \boldsymbol{\epsilon}_{\boldsymbol{\lambda}}\end{aligned} \tag{S7}$$

$$\Rightarrow \nabla_{\mu_\lambda}\left[-\frac{1}{2}\boldsymbol{\epsilon}_{\boldsymbol{\lambda}}^T \boldsymbol{\Pi}_{\boldsymbol{\lambda}} \boldsymbol{\epsilon}_{\boldsymbol{\lambda}}\right] = -\partial_{\mu_\lambda}(\boldsymbol{\epsilon}_{\boldsymbol{\lambda}})^{\mathrm{T}} \boldsymbol{\Pi}_{\boldsymbol{\lambda}} \boldsymbol{\epsilon}_{\boldsymbol{\lambda}} = -\boldsymbol{\Pi}_{\boldsymbol{\lambda}} \boldsymbol{\epsilon}_{\boldsymbol{\lambda}}$$

The second term is:

$$\begin{aligned}\left(\nabla_{\mu_\lambda}\left[-\frac{1}{2}\ln\left|\boldsymbol{\Pi}_{\mathbf{y}}^{-\mathbf{1}}\right|\right]\right)_i &= -\frac{1}{2}\partial_{\mu_{\lambda_i}}\left[\ln\left|\boldsymbol{\Pi}_{\mathbf{y}}^{-\mathbf{1}}\right|\right] \\ &= -\frac{1}{2}\partial_{\mu_{\lambda_i}}\left[-\ln\left|\boldsymbol{\Pi}_{\mathbf{y}}\right|\right] \\ &= \frac{1}{2}tr\left(\boldsymbol{\Pi}_{\boldsymbol{y}}^{-\mathbf{1}}\boldsymbol{P}_{\boldsymbol{i}}\right) \\ &= \frac{1}{2}tr\left(\boldsymbol{P}_{\boldsymbol{i}}\boldsymbol{\Pi}_{\boldsymbol{y}}^{-\mathbf{1}}\right)\end{aligned} \tag{S8}$$

Where $\boldsymbol{P_i} = \partial_{\mu_{\lambda_i}}(\boldsymbol{\Pi_y}) = \partial_{\mu_{\lambda_i}\mu_{\lambda_i}}(\boldsymbol{\Pi_y}) = \exp(\lambda_i)\boldsymbol{\Pi_i}$ and the third line used the identity $\partial(\ln|\boldsymbol{X}|) = tr(\boldsymbol{X}^{-1}\partial\boldsymbol{X})$ – see Eq 43 of (Petersen and Pedersen, 2008). Finally, the third term is:

$$\begin{aligned}\left(\nabla_{\mu_\lambda}\left[-\frac{1}{2}\boldsymbol{\epsilon}_{\boldsymbol{y}}^T \boldsymbol{\Pi}_{\mathbf{y}} \boldsymbol{\epsilon}_{\boldsymbol{y}}\right]\right)_i &= -\frac{1}{2}\sum_j \sum_k \partial_{\mu_{\lambda_i}}\left(\epsilon_{y_j}\epsilon_{y_k}\Pi_{y_{jk}}\right) \\ = -\frac{1}{2}\sum_j \sum_k \partial_{\mu_{\lambda_i}}\left(\epsilon_{y_j}\right)\epsilon_{y_k}\Pi_{y_{jk}} &- \frac{1}{2}\sum_j \sum_k \epsilon_{y_j}\partial_{\mu_{\lambda_i}}\left(\epsilon_{y_k}\right)\Pi_{y_{jk}} - \frac{1}{2}\sum_j \sum_k \epsilon_{y_j}\epsilon_{y_k}\partial_{\mu_{\lambda_i}}\left(\Pi_{y_{jk}}\right) \\ = -\frac{1}{2}\boldsymbol{\epsilon}_{\boldsymbol{y}}^{\boldsymbol{T}}\boldsymbol{P}_{\boldsymbol{i}}\boldsymbol{\epsilon}_{\boldsymbol{y}} & \\ = -\frac{1}{2}tr\left(\boldsymbol{P}_{\boldsymbol{i}}\left(\boldsymbol{\epsilon}_{y}\boldsymbol{\epsilon}_{y}^{T}\right)\right) &\end{aligned} \tag{S9}$$

Assembling these three terms:

$$\begin{aligned}\left(\nabla_{\mu_\lambda} \ln P(\boldsymbol{y}, \boldsymbol{\mu_\beta}, \boldsymbol{\mu_\lambda})\right)_{\mathrm{i}} &= -\partial_{\mu_{\lambda_i}}(\boldsymbol{\epsilon}_{\boldsymbol{\lambda}})^{\mathrm{T}} \boldsymbol{\Pi}_{\boldsymbol{\lambda}} \boldsymbol{\epsilon}_{\boldsymbol{\lambda}} + \frac{1}{2}tr\left(\partial_{\mu_{\lambda_i}}\left[\boldsymbol{\Pi}_{\mathbf{y}}\right]\boldsymbol{\Pi}_{\boldsymbol{y}}^{-\mathbf{1}}\right) - \frac{1}{2}\boldsymbol{\epsilon}_{\boldsymbol{y}}^{\boldsymbol{T}}\partial_{\mu_{\lambda_i}}(\boldsymbol{\Pi}_{\mathbf{y}})\boldsymbol{\epsilon}_{\boldsymbol{y}} \\ &= -\partial_{\mu_{\lambda_i}}(\boldsymbol{\epsilon}_{\boldsymbol{\lambda}})^{\mathrm{T}} \boldsymbol{\Pi}_{\boldsymbol{\lambda}} \boldsymbol{\epsilon}_{\boldsymbol{\lambda}} + \frac{1}{2}tr\left(\boldsymbol{P}_{\boldsymbol{i}}\left(\boldsymbol{\Pi}_{\boldsymbol{y}}^{-\mathbf{1}} - \left(\boldsymbol{\epsilon}_{y}\boldsymbol{\epsilon}_{y}^{T}\right)\right)\right)\end{aligned} \tag{S10}$$

1.3 Second derivatives

The second partial derivatives of the log joint with respect to the parameters are arranged in the Hessian matrix $\boldsymbol{H_\beta}$ where $(\boldsymbol{H_\beta})_{i,j} = \partial_{\mu_{\beta_i}\mu_{\beta_j}} \ln P(y, \mu_\beta, \lambda)$. This matrix is derived as follows:

$$
\begin{aligned}
\boldsymbol{H}_{\boldsymbol{\beta}} = \partial_{\mu_\beta \mu_\beta} \ln P(\boldsymbol{y}, \boldsymbol{\mu}_{\boldsymbol{\beta}}, \boldsymbol{\mu}_{\boldsymbol{\lambda}}) &= \partial_{\mu_\beta}\left(g'(\boldsymbol{\mu})^T \boldsymbol{\Pi}_{\boldsymbol{y}} \boldsymbol{\epsilon}_{\boldsymbol{y}}\right) - \partial_{\mu_\beta}\left(\boldsymbol{\Pi}_{\boldsymbol{\beta}} \boldsymbol{\epsilon}_{\boldsymbol{\beta}}\right) \\
&= \partial_{\mu_\beta}\left(g'(\boldsymbol{\mu})^T \boldsymbol{\Pi}_{\boldsymbol{y}} \boldsymbol{\epsilon}_{\boldsymbol{y}}\right) - \boldsymbol{\Pi}_{\boldsymbol{\beta}} \\
&= \partial_{\mu_\beta}\left(g'(\boldsymbol{\mu})\right)^T \boldsymbol{\Pi}_{\boldsymbol{y}} \boldsymbol{\epsilon}_{\boldsymbol{y}} + g'(\boldsymbol{\mu})^T \partial_{\mu_\beta}(\boldsymbol{\Pi}_{\boldsymbol{y}}) \boldsymbol{\epsilon}_{\boldsymbol{y}} + g'(\boldsymbol{\mu})^T \boldsymbol{\Pi}_{\boldsymbol{y}} \partial_{\mu_\beta}(\boldsymbol{\epsilon}_{\boldsymbol{y}}) - \boldsymbol{\Pi}_{\boldsymbol{\beta}} \\
&\approx -\boldsymbol{J}_{\boldsymbol{g}}^{\boldsymbol{T}} \boldsymbol{\Pi}_{\boldsymbol{y}} \boldsymbol{J}_{\boldsymbol{g}} - \boldsymbol{\Pi}_{\boldsymbol{\beta}}
\end{aligned}
\tag{S11}
$$

Where $g'(\boldsymbol{\mu})$ and $g''(\boldsymbol{\mu})$ are shorthand for the first and second derivatives respectively. In SPM, terms that depend on the second derivative $g''(\boldsymbol{\mu})$ are dropped, under the assumption that the model is only weakly non-linear. The second derivatives of the log joint with respect to the hyperparameters are arranged on the leading diagonal of the Hessian matrix $\boldsymbol{H}_{\boldsymbol{\lambda}}$:

$$
\begin{aligned}
(\boldsymbol{H}_{\boldsymbol{\lambda}})_{i,i} &= \partial_{\mu_{\lambda_i} \mu_{\lambda_i}} \ln P(\boldsymbol{y}, \boldsymbol{\mu}_{\boldsymbol{\beta}}, \boldsymbol{\mu}_{\boldsymbol{\lambda}_i}) \\
&= -\partial_{\mu_{\lambda_i}}\left(\partial_{\mu_{\lambda_i}}(\boldsymbol{\epsilon}_{\boldsymbol{\lambda}})^{\mathrm{T}} \boldsymbol{\Pi}_{\boldsymbol{\lambda}} \boldsymbol{\epsilon}_{\boldsymbol{\lambda}}\right) - \partial_{\mu_{\lambda_i}}\left(\frac{1}{2} \boldsymbol{\epsilon}_{\boldsymbol{y}}^{T} \boldsymbol{P}_{\boldsymbol{i}} \boldsymbol{\epsilon}_{\boldsymbol{y}}\right) \\
&= -\partial_{\mu_{\lambda_i}}(\boldsymbol{\epsilon}_{\boldsymbol{\lambda}})^{\mathrm{T}} \boldsymbol{\Pi}_{\boldsymbol{\lambda}} \partial_{\mu_{\lambda_i}}(\boldsymbol{\epsilon}_{\boldsymbol{\lambda}}) - \frac{1}{2} \boldsymbol{\epsilon}_{\boldsymbol{y}}^{\boldsymbol{T}} \boldsymbol{P}_{\boldsymbol{i}} \boldsymbol{\epsilon}_{\boldsymbol{y}} \\
&= -(\boldsymbol{\Pi}_{\boldsymbol{\lambda}})_{i,i} - \frac{1}{2} \boldsymbol{\epsilon}_{\boldsymbol{y}}^{\boldsymbol{T}} \boldsymbol{P}_{\boldsymbol{i}} \boldsymbol{\epsilon}_{\boldsymbol{y}}
\end{aligned}
\tag{S12}
$$

---

2. Derivation of Eq 38

Eq. 38 is the fundamental premise of mean-field variational Bayes, and the derivation is as follows. The free energy under the mean-field approximation is:

$$
\begin{aligned}
F[Q(\boldsymbol{\theta})] &= E_{Q(\boldsymbol{\beta})Q(\boldsymbol{\lambda})}\left[\ln \frac{P(\boldsymbol{y}, \boldsymbol{\beta}, \boldsymbol{\lambda})}{Q(\boldsymbol{\beta})Q(\boldsymbol{\lambda})}\right] \\
&= E_{Q(\boldsymbol{\beta})Q(\boldsymbol{\lambda})}[\ln P(\boldsymbol{y}, \boldsymbol{\beta}, \boldsymbol{\lambda})] - E_{Q(\boldsymbol{\beta})}[\ln Q(\boldsymbol{\beta})] - E_{Q(\boldsymbol{\lambda})}[\ln Q(\boldsymbol{\lambda})]
\end{aligned}
\tag{S13}
$$

Next, we'll re-express the free energy, expanding the expected values and separating out terms relating to $Q(\boldsymbol{\beta})$, treating any other terms as constant.

$$
\begin{aligned}
F[Q(\boldsymbol{\theta})] &= \int_{\boldsymbol{\beta}}\int_{\boldsymbol{\lambda}} Q(\boldsymbol{\beta})Q(\boldsymbol{\lambda})[\ln P(\boldsymbol{y},\boldsymbol{\beta},\boldsymbol{\lambda}) - \ln Q(\boldsymbol{\beta}) - \ln Q(\boldsymbol{\lambda})]d\boldsymbol{\beta}d\boldsymbol{\lambda} \\
&= \int_{\boldsymbol{\beta}}\int_{\boldsymbol{\lambda}} Q(\boldsymbol{\beta})Q(\boldsymbol{\lambda})\ln P(\boldsymbol{y},\boldsymbol{\beta},\boldsymbol{\lambda})\, d\boldsymbol{\beta}d\boldsymbol{\lambda} - \int_{\boldsymbol{\beta}}\int_{\boldsymbol{\lambda}} Q(\boldsymbol{\beta})Q(\boldsymbol{\lambda})[\ln Q(\boldsymbol{\beta}) + \ln Q(\boldsymbol{\lambda})]d\boldsymbol{\beta}d\boldsymbol{\lambda} \\
&= \int_{\boldsymbol{\beta}} Q(\boldsymbol{\beta})\left(\int_{\boldsymbol{\lambda}} Q(\boldsymbol{\lambda})\ln P(\boldsymbol{y},\boldsymbol{\beta},\boldsymbol{\lambda})\, d\boldsymbol{\lambda}\right)d\boldsymbol{\beta} - \int_{\boldsymbol{\beta}} Q(\boldsymbol{\beta})\ln Q(\boldsymbol{\beta})d\boldsymbol{\beta}\underbrace{\left(\int_{\boldsymbol{\lambda}} Q(\boldsymbol{\lambda})d\boldsymbol{\lambda}\right)}_{1} \\
&\qquad - \underbrace{\int_{\boldsymbol{\beta}} Q(\boldsymbol{\beta})d\boldsymbol{\beta}}_{1}\left(\int_{\boldsymbol{\lambda}} Q(\boldsymbol{\lambda})\ln Q(\boldsymbol{\lambda})\, d\boldsymbol{\lambda}\right) \\
&= \int_{\boldsymbol{\beta}} \big[Q(\boldsymbol{\beta})E_{Q(\lambda)}[\ln P(\boldsymbol{y},\boldsymbol{\beta},\boldsymbol{\lambda})] - Q(\boldsymbol{\beta})\ln Q(\boldsymbol{\beta})\big]d\boldsymbol{\beta} - E_{Q(\lambda)}\ln Q(\boldsymbol{\lambda}) \\
&= \int_{\boldsymbol{\beta}} \big[Q(\boldsymbol{\beta})E_{Q(\lambda)}[\ln P(\boldsymbol{y},\boldsymbol{\beta},\boldsymbol{\lambda})] - Q(\boldsymbol{\beta})\ln Q(\boldsymbol{\beta})\big]d\boldsymbol{\beta} + c
\end{aligned}
\tag{S14}
$$

For convenience we will label the integral within the free energy $I[Q(\boldsymbol{\beta})]$ and its integrand $\mathcal{L}[Q(\boldsymbol{\beta})]$:

$$
I[Q(\boldsymbol{\beta})] = \int_{\boldsymbol{\beta}} \underbrace{Q(\boldsymbol{\beta})E_{Q(\lambda)}[\ln P(\boldsymbol{y},\boldsymbol{\beta},\boldsymbol{\lambda})] - Q(\boldsymbol{\beta})\ln Q(\boldsymbol{\beta})}_{\mathcal{L}[Q(\boldsymbol{\beta})]}\, d\boldsymbol{\beta}
\tag{S15}
$$

The functional derivative of the free energy with respect to the parameters is therefore:

$$
\delta_{Q(\boldsymbol{\beta})}F[Q(\boldsymbol{\beta})] = \delta_{Q(\boldsymbol{\beta})}\big[I[Q(\boldsymbol{\beta})]\big]
\tag{S16}
$$

This functional derivative can be simplified using the Euler-Lagrange equation, which states that for a functional $\mathcal{L}$ of a function $f(x)$, with the integral $I[f] = \int \mathcal{L}(f)dx$, we can set its functional derivative to zero, which implies the partial derivative of the integrand of the functional $\mathcal{L}$ is also zero:

$$
\frac{\delta I}{\delta f} = 0 \Rightarrow \frac{\partial \mathcal{L}}{\partial f} = 0
\tag{S17}
$$

Applying this to the integral above:

$$
\begin{aligned}
\delta_{Q(\boldsymbol{\beta})}I[Q(\boldsymbol{\beta})] = 0 &\Rightarrow \partial_{Q(\boldsymbol{\beta})}\big[Q(\boldsymbol{\beta})E_{Q(\lambda)}[\ln P(\boldsymbol{y},\boldsymbol{\beta},\boldsymbol{\lambda})] - Q(\boldsymbol{\beta})\ln Q(\boldsymbol{\beta})\big] = 0 \\
&\Rightarrow\ Q(\boldsymbol{\beta})\underbrace{\partial_{Q(\boldsymbol{\beta})}E_{Q(\lambda)}[\ln P(\boldsymbol{y},\boldsymbol{\beta},\boldsymbol{\lambda})]}_{0} + \underbrace{\partial_{Q(\boldsymbol{\beta})}Q(\boldsymbol{\beta})}_{1}E_{Q(\lambda)}[\ln P(\boldsymbol{y},\boldsymbol{\beta},\boldsymbol{\lambda})] \\
&\quad - (1 + \ln Q(\boldsymbol{\beta})) = 0 \\
&\Rightarrow E_{Q(\lambda)}[\ln P(\boldsymbol{y},\boldsymbol{\beta},\boldsymbol{\lambda})] - \ln Q(\boldsymbol{\beta}) - 1 = 0
\end{aligned}
\tag{S18}
$$

Therefore, up to a constant, the variation of the free energy with respect to $Q(\boldsymbol{\beta})$ is (Eq 37):

$$
\delta_{Q(\boldsymbol{\beta})}F[Q(\boldsymbol{\beta})] = -\ln Q(\boldsymbol{\beta}) + E_{Q(\lambda)}[\ln P(\boldsymbol{y},\boldsymbol{\beta},\boldsymbol{\lambda})]
$$

And by re-arranging and taking the exponential, the posteriors that maximize the free energy are (Eq 38):

$$
Q(\boldsymbol{\beta}) \propto \exp\big(E_{Q(\lambda)}[\ln P(\boldsymbol{y},\boldsymbol{\beta},\boldsymbol{\lambda})]\big)
$$

A similar expression can be derived for $\delta_{Q(\lambda)}F$ and $Q(\boldsymbol{\lambda})$.

---

3. Derivation of Eq 40

The gradient ascent update equations are derived as follows. From Eq 38:

$$\begin{aligned}
\ln Q(\boldsymbol{\beta}) &\propto E_{Q(\lambda)}[\ln P(\boldsymbol{y},\boldsymbol{\beta},\boldsymbol{\lambda})] = \ln P(\boldsymbol{y},\boldsymbol{\beta},\boldsymbol{\mu_\lambda}) + \frac{1}{2}tr(\boldsymbol{\Sigma_\lambda}\,\partial_{\mu_\lambda\mu_\lambda}\ln P(\boldsymbol{y},\boldsymbol{\beta},\boldsymbol{\mu_\lambda})) \\
\ln Q(\boldsymbol{\lambda}) &\propto E_{Q(\boldsymbol{\beta})}[\ln P(\boldsymbol{y},\boldsymbol{\beta},\boldsymbol{\lambda})] = \ln P(\boldsymbol{y},\boldsymbol{\mu_\beta},\boldsymbol{\lambda}) + \frac{1}{2}tr\left(\boldsymbol{\Sigma_\beta}\partial_{\mu_\beta\mu_\beta}\ln P(\boldsymbol{y},\boldsymbol{\mu_\beta},\boldsymbol{\lambda})\right)
\end{aligned} \qquad \textit{(S19)}$$

The algorithm alternates between updating the parameters to maximize $\ln Q(\boldsymbol{\beta})$ and the hyperparameters to maximize $\ln Q(\boldsymbol{\lambda})$. The update for each iteration is:

$$\begin{aligned}
\boldsymbol{\Delta\mu_\beta} &= \nabla_{\mu_\beta}E_{Q(\lambda)}[\ln P(\boldsymbol{y},\boldsymbol{\mu_\beta},\boldsymbol{\lambda})] \\
&= \nabla_{\mu_\beta}\ln P(\boldsymbol{y},\boldsymbol{\mu_\beta},\boldsymbol{\mu_\lambda}) + \nabla_{\mu_\beta}\frac{1}{2}tr(\boldsymbol{\Sigma_\lambda}\,\partial_{\mu_\lambda\mu_\lambda}\ln P(\boldsymbol{y},\boldsymbol{\mu_\beta},\boldsymbol{\mu_\lambda})) \\
&= \nabla_{\mu_\beta}\ln P(\boldsymbol{y},\boldsymbol{\mu_\beta},\boldsymbol{\mu_\lambda}) + \frac{1}{2}\nabla_{\mu_\beta}tr(\boldsymbol{\Sigma_\lambda}\,\boldsymbol{H_\lambda})
\end{aligned}$$

$$\begin{aligned}
\nabla_{\mu_\beta}\ln P(\boldsymbol{y},\boldsymbol{\mu_\beta},\boldsymbol{\mu_\lambda}) &= \mathbf{J}_g^T\boldsymbol{\Pi_y\epsilon_y} - \boldsymbol{\Pi_\beta\epsilon_\beta} \\
\left(\nabla_{\mu_\beta}tr(\boldsymbol{\Sigma_\lambda}\,\boldsymbol{H_\lambda})\right)_i &= \partial_{\mu_{\beta_i}}tr(\boldsymbol{\Sigma_\lambda}\,\boldsymbol{H_\lambda}) \\
&= \partial_{\mu_{\beta_i}}\sum_{j=1}^{h}(\boldsymbol{\Sigma_\lambda})_{jj}\left[-(\boldsymbol{\Pi_\lambda})_{j,j} - \left[\frac{1}{2}\boldsymbol{\epsilon_y^T P}_j\boldsymbol{\epsilon_y}\right]\right] \\
&= -\partial_{\mu_{\beta_i}}\sum_{j=1}^{h}(\boldsymbol{\Sigma_\lambda})_{jj}(\boldsymbol{\Pi_\lambda})_{j,j} - \frac{1}{2}\partial_{\mu_{\beta_i}}\sum_{j=1}^{h}(\boldsymbol{\Sigma_\lambda})_{jj}[\boldsymbol{\epsilon_y^T P}_j\boldsymbol{\epsilon_y}] \\
&= -\frac{1}{2}\sum_{j=1}^{h}\partial_{\mu_{\beta_i}}\left[(\boldsymbol{\Sigma_\lambda})_{jj}[\boldsymbol{\epsilon_y^T P}_j\boldsymbol{\epsilon_y}]\right] \\
&= -\frac{1}{2}\sum_{j=1}^{h}(\boldsymbol{\Sigma_\lambda})_{jj}\,\partial_{\mu_{\beta_i}}[\boldsymbol{\epsilon_y^T P}_j\boldsymbol{\epsilon_y}] \\
&= -\frac{1}{2}\sum_{j=1}^{h}(\boldsymbol{\Sigma_\lambda})_{jj}\left[\partial_{\mu_{\beta_i}}[\boldsymbol{\epsilon_y^T}]\boldsymbol{P}_j\boldsymbol{\epsilon_y} + \boldsymbol{\epsilon_y^T P_j}\,\partial_{\mu_{\beta_i}}[\boldsymbol{\epsilon_y}]\right] \\
&= \sum_{j=1}^{h}(\boldsymbol{\Sigma_\lambda})_{jj}\,\partial_{\mu_{\beta_i}}[g(\boldsymbol{\mu_\beta})]\boldsymbol{P}_j\boldsymbol{\epsilon_y}
\end{aligned} \qquad \textit{(S20)}$$

$$\Rightarrow \boldsymbol{\Delta\mu_\beta} = \mathbf{J}_g^T\boldsymbol{\Pi_y\epsilon_y} - \boldsymbol{\Pi_\beta\epsilon_\beta} + \sum_{j=1}^{h}(\boldsymbol{\Sigma_\lambda})_{jj}\boldsymbol{J_g^T P}_j\boldsymbol{\epsilon_y}$$

Similarly, the update for the hyperparameters is:

$$
\begin{aligned}
(\Delta\mu_{\lambda})_i &= \partial_{\mu_{\lambda_i}} E_{Q(\beta)}[\ln P(\boldsymbol{y}, \boldsymbol{\beta}, \boldsymbol{\mu_\lambda})] \\
&= \partial_{\mu_{\lambda_i}} \ln P(\boldsymbol{y}, \boldsymbol{\mu_\beta}, \boldsymbol{\lambda}) + \partial_{\mu_{\lambda_i}} \frac{1}{2} tr\left(\boldsymbol{\Sigma_\beta} \partial_{\mu_\beta \mu_\beta} \ln P(\boldsymbol{y}, \boldsymbol{\mu_\beta}, \boldsymbol{\lambda})\right) \\
&= \partial_{\mu_{\lambda_i}} \ln P(\boldsymbol{y}, \boldsymbol{\mu_\beta}, \boldsymbol{\lambda}) + \partial_{\mu_{\lambda_i}} \frac{1}{2} tr(\boldsymbol{\Sigma_\beta H_\beta}) \\
&= \partial_{\mu_{\lambda_i}} \ln P(\boldsymbol{y}, \boldsymbol{\mu_\beta}, \boldsymbol{\lambda}) + \partial_{\mu_{\lambda_i}} \frac{1}{2} tr(-\boldsymbol{\Sigma_\beta J_g^T \Pi_y J_g} - \boldsymbol{\Sigma_\beta \Pi_\beta}) \\
&= \partial_{\mu_{\lambda_i}} \ln P(\boldsymbol{y}, \boldsymbol{\mu_\beta}, \boldsymbol{\lambda}) - \frac{1}{2} tr\left(\partial_{\mu_{\lambda_i}}[\boldsymbol{\Sigma_\beta J_g^T \Pi_y J_g}]\right) \\
&= \partial_{\mu_{\lambda_i}} \ln P(\boldsymbol{y}, \boldsymbol{\mu_\beta}, \boldsymbol{\lambda}) - \frac{1}{2} tr(\boldsymbol{\Sigma_\beta J_g^T P_i J_g}) \\
&= -\partial_{\mu_{\lambda_i}} (\boldsymbol{\epsilon_\lambda})^{\mathrm{T}} \boldsymbol{\Pi_\lambda \epsilon_\lambda} + \frac{1}{2} tr(\boldsymbol{P_i \Pi_y^{-1}}) - \frac{1}{2} \boldsymbol{\epsilon_y^T P_i \epsilon_y} - \frac{1}{2} tr(\boldsymbol{\Sigma_\beta J_g^T P_i J_g})
\end{aligned}
\tag{S21}
$$

---

4. Derivation of Eq 41

The expression required to compute the posterior covariance over parameters is:

$$
\begin{aligned}
\partial_{\mu_\beta \mu_\beta} E_{Q(\lambda)}[\ln P(\boldsymbol{y}, \boldsymbol{\mu_\beta}, \boldsymbol{\lambda})] &= \partial_{\mu_\beta}[\mathbf{J}_g^T \boldsymbol{\Pi_y \epsilon_y}] - \partial_{\mu_\beta}[\boldsymbol{\Pi_\beta \epsilon_\beta}] + \partial_{\mu_\beta}\left[\sum_{j=1}^{h} (\boldsymbol{\Sigma_\lambda})_{jj} \boldsymbol{J_g^T P}_j \boldsymbol{\epsilon_y}\right] \\
&= \partial_{\mu_\beta}[\boldsymbol{J_g^T}] \boldsymbol{\Pi_y \epsilon_y} + \mathbf{J}_g^T \boldsymbol{\Pi_y} \partial_{\mu_\beta}[\boldsymbol{\epsilon_y}] - \boldsymbol{\Pi_\beta} + \sum_{j=1}^{h} \partial_{\mu_\beta}[(\boldsymbol{\Sigma_\lambda})_{jj} \boldsymbol{J_g^T P}_j \boldsymbol{\epsilon_y}] \\
&\approx -\mathbf{J}_g^T \boldsymbol{\Pi_y J_g} - \boldsymbol{\Pi_\beta} - \sum_{j=1}^{h} (\boldsymbol{\Sigma_\lambda})_{jj} \boldsymbol{J_g^T P}_j \boldsymbol{J_g} \\
&\approx -\mathbf{J}_g^T \boldsymbol{\Pi_y J_g} - \boldsymbol{\Pi_\beta}
\end{aligned}
\tag{S22}
$$

For the hyperparameter this is a little more involved:

$$
\begin{aligned}
&\partial_{\mu_{\lambda_i} \mu_{\lambda_i}} E_{Q(\beta)}[\ln P(\boldsymbol{y}, \boldsymbol{\beta}, \boldsymbol{\mu_\lambda})] \\
&= -\partial_{\mu_{\lambda_i}}\left[\partial_{\mu_{\lambda_i}} (\boldsymbol{\epsilon_\lambda})^{\mathrm{T}} \boldsymbol{\Pi_\lambda \epsilon_\lambda}\right] + \frac{1}{2} \partial_{\mu_{\lambda_i}}[tr(\boldsymbol{P_i \Pi_y^{-1}})] - \frac{1}{2} \partial_{\mu_{\lambda_i}}[\boldsymbol{\epsilon_y^T P_i \epsilon_y}] \\
&- \frac{1}{2} \partial_{\mu_{\lambda_i}} tr(\boldsymbol{\Sigma_\beta J_g^T P_i J_g})
\end{aligned}
\tag{S23}
$$

Tackling each of the four terms in Eq S23, first:

$$
-\partial_{\mu_{\lambda_i}}\left[\partial_{\mu_{\lambda_i}} (\boldsymbol{\epsilon_\lambda})^{\mathrm{T}} \boldsymbol{\Pi_\lambda \epsilon_\lambda}\right] = -\partial_{\mu_{\lambda_i}} (\boldsymbol{\epsilon_\lambda})^{\mathrm{T}} \boldsymbol{\Pi_\lambda} \partial_{\mu_{\lambda_i}} (\boldsymbol{\epsilon_\lambda}) = -(\boldsymbol{\Pi_\lambda})_{ii}
\tag{S24}
$$

Second:

$$\frac{1}{2}\partial_{\mu_{\lambda_i}}\left[tr(\boldsymbol{P}_i\boldsymbol{\Sigma}_y)\right] = \frac{1}{2}tr\left(\partial_{\mu_{\lambda_i}}[\boldsymbol{P}_i\boldsymbol{\Sigma}_y]\right)$$
$$= \frac{1}{2}tr\left(\boldsymbol{P}_i\boldsymbol{\Sigma}_y + \boldsymbol{P}_i\,\partial_{\mu_{\lambda_i}}[\boldsymbol{\Sigma}_y]\right)$$
$$= \frac{1}{2}tr\left(\boldsymbol{P}_i\boldsymbol{\Sigma}_y + \boldsymbol{P}_i(-\boldsymbol{\Sigma}_y\boldsymbol{P}_i\boldsymbol{\Sigma}_y)\right) \tag{S25}$$
$$= \frac{1}{2}tr\left(\boldsymbol{P}_i\boldsymbol{\Sigma}_y - \boldsymbol{P}_i\boldsymbol{\Sigma}_y\boldsymbol{P}_i\boldsymbol{\Sigma}_y\right)$$

Where $\boldsymbol{\Sigma}_y = \boldsymbol{\Pi}_y^{-1}$. Third:

$$-\frac{1}{2}\partial_{\mu_{\lambda_i}}\left[\boldsymbol{\epsilon}_y^T\boldsymbol{P}_i\boldsymbol{\epsilon}_y\right] = -\frac{1}{2}\boldsymbol{\epsilon}_y^T\boldsymbol{P}_i\boldsymbol{\epsilon}_y \tag{S26}$$

And fourth:

$$-\frac{1}{2}\partial_{\mu_{\lambda_i}}tr\left(\boldsymbol{\Sigma}_\beta\boldsymbol{J}_g^T\boldsymbol{P}_i\boldsymbol{J}_g\right) = -\frac{1}{2}tr\left(\partial_{\mu_{\lambda_i}}[\boldsymbol{\Sigma}_\beta\boldsymbol{J}_g^T\boldsymbol{P}_i\boldsymbol{J}_g]\right) = -\frac{1}{2}tr\left(\boldsymbol{\Sigma}_\beta\boldsymbol{J}_g^T\boldsymbol{P}_i\boldsymbol{J}_g\right) \tag{S27}$$

Thus:

$$\partial_{\mu_{\lambda_i}\mu_{\lambda_i}}E_{Q(\beta)}[\ln P(\boldsymbol{y},\boldsymbol{\beta},\boldsymbol{\lambda})] = -(\boldsymbol{\Pi}_\lambda)_{ii} + \frac{1}{2}tr\left(\boldsymbol{P}_i\boldsymbol{\Sigma}_y - \boldsymbol{P}_i\boldsymbol{\Sigma}_y\boldsymbol{P}_i\boldsymbol{\Sigma}_y\right) - \frac{1}{2}\boldsymbol{\epsilon}_y^T\boldsymbol{P}_i\boldsymbol{\epsilon}_y - \frac{1}{2}tr\left(\boldsymbol{\Sigma}_\beta\boldsymbol{J}_g^T\boldsymbol{P}_i\boldsymbol{J}_g\right) \tag{S28}$$

---

5. Derivation of Eq 43

This is an application of the local linearization scheme of Ozaki (1985), for approximating the integration of a continuous function via discrete steps. For one or more model parameters $\mu(t)$ at time t we have the gradient ascent:

$$\dot{\mu}(t) = f(\mu(t)) \tag{S29}$$

In the context of the VL scheme, the function on the right-hand side is $f\left(\mu_\beta(t)\right) = \nabla_{\mu_\beta}\left[I_\beta(\mu_\beta[t])\right]$ for updating the parameters and $f(\mu_\lambda(t)) = \nabla_{\mu_\lambda}[I_\lambda(\mu_\lambda[t])]$ for updating the hyperparameters. Taking the time derivative again, we obtain:

$$\ddot{\mu}(t) = J[f(\mu(t))]\dot{\mu}(t)$$
$$J(\mu(t)) = \partial_{\mu(t)}f(\mu(t)) \tag{S30}$$

If we assume that $f(\mu(t))$ is linear over a short interval $t \in [0, \Delta t]$, we can replace J with the Jacobian at the origin of the dynamical system, $J_0 = \partial_{\mu(0)}f(\mu(0))$. Thus:

$$\ddot{\mu}(t) = J_0\,\dot{\mu}(t) \tag{S31}$$

Integrating over the short interval $t \in [0, \tau]$, where $\tau \in [0, \Delta t]$, gives:

$$\dot{\mu}(\tau) = \dot{\mu}(0)\exp(J_0\tau) \tag{S32}$$

Integrating again, this time over $\tau \in [0, \Delta t]$, gives:

$$\mu(\Delta t) = \mu(0) + J_0^{-1}(\exp[J_0\Delta t] - I) f(\mu(0)) \tag{S33}$$

---

7. Derivation of Eq 56

The log Bayes factor for model m_1 relative to m_2 is converted to a posterior probability in favour of m_1, under equal priors for both models, via Bayes rule:

$$\begin{aligned} P(m_1|y) &= \frac{P(y|m_1)P(m)}{P(y)} \\ &= \frac{P(y|m_1)P(m)}{P(y|m_1)P(m) + P(y|m_2)P(m)} \\ &= \frac{P(y|m_1)}{P(y|m_1) + P(y|m_2)} \\ &= \frac{1}{1 + \frac{P(y|m_2)}{P(y|m_1)}} \\ &= \frac{1}{1 + B_2} \\ &= \frac{1}{1 + \exp[\ln B_2]} \\ &= \frac{1}{1 + \exp[-\ln B_1]} \end{aligned} \tag{S34}$$

Where $P(m) = P(m_1) = P(m_2)$ and B_x is the Bayes factor in favour of model x. We have written this in such a way to demonstrate that the posterior probability a softmax function of the log Bayes factor.

8. Supplementary references

OZAKI, T. 1985. Non-linear time series models and dynamical systems. *Handbook of statistics.*
PETERSEN, K. B. & PEDERSEN, M. S. 2008. The matrix cookbook. *Technical University of Denmark,* 7, 510.
